# Longitudinal in vivo imaging of acute neuropathology in a monkey model of Ebola virus infection

**DOI:** 10.1038/s41467-021-23088-x

**Published:** 2021-05-17

**Authors:** William Schreiber-Stainthorp, Jeffrey Solomon, Ji Hyun Lee, Marcelo Castro, Swati Shah, Neysha Martinez-Orengo, Rebecca Reeder, Dragan Maric, Robin Gross, Jing Qin, Katie R. Hagen, Reed F. Johnson, Dima A. Hammoud

**Affiliations:** 1 Hammoud Laboratory, Center for Infectious Disease Imaging (CIDI), Radiology and Imaging Sciences, Clinical Center, National Institutes of Health (NIH), Bethesda, MD USA; 2 Clinical Monitoring Research Program Directorate, Frederick National Laboratory for Cancer Research, Frederick, MD USA; 3 Integrated Research Facility, National Institute of Allergy and Infectious Diseases, National Institutes of Health, Frederick, MD USA; 4 Flow and Imaging Cytometry Core Facility, National Institute of Neurological Disorders and Stroke (NINDS), National Institutes of Health, Bethesda, MD USA; 5 Biostatistics Research Branch, National Institute of Allergy and Infectious Diseases, National Institutes of Health, Rockville, MD USA; 6 Emerging Viral Pathogens Section, National Institute of Allergy and Infectious Diseases, National Institutes of Health, Frederick, MD USA

**Keywords:** Ebola virus, Cell death in the nervous system

## Abstract

Ebola virus (EBOV) causes neurological symptoms yet its effects on the central nervous system (CNS) are not well-described. Here, we longitudinally assess the acute effects of EBOV on the brain, using quantitative MR-relaxometry, 18F-Fluorodeoxyglucose PET and immunohistochemistry in a monkey model. We report blood–brain barrier disruption, likely related to high cytokine levels and endothelial viral infection, with extravasation of fluid, Gadolinium-based contrast material and albumin into the extracellular space. Increased glucose metabolism is also present compared to the baseline, especially in the deep gray matter and brainstem. This regional hypermetabolism corresponds with mild neuroinflammation, sporadic neuronal infection and apoptosis, as well as increased GLUT3 expression, consistent with increased neuronal metabolic demands. Neuroimaging changes are associated with markers of disease progression including viral load and cytokine/chemokine levels. Our results provide insight into the pathophysiology of CNS involvement with EBOV and may help assess vaccine/treatment efficacy in real time.

## Abstract (web summary)

The neurological effects of Ebola disease in the acute stage are not well-described. Here, the authors use longitudinal in vivo neuroimaging and immunohistochemistry to assess pathological changes in the central nervous system in a non-human primate model of Ebola virus infection.

## Introduction

The 2014–2016 Ebola virus (EBOV) outbreak in West Africa infected over 28,000 people and caused >11,000 deaths^1^. This was the largest documented outbreak of EBOV, although the disease continued to wreak damage: another outbreak, which began in 2018 infected over 3400 people in the Democratic Republic of Congo (DRC) with a case fatality ratio of 66% (WHO Situation Report 06/24/2020). The toll of EBOV infection however is not limited to the acute consequences: survivors of the disease have struggled to reintegrate into society and have shown elevated levels of disability^2,3^. This phenomenon has been referred to as “post-Ebolavirus disease syndrome” (PEVDS), and it encompasses a range of symptoms including extreme fatigue, difficulty sleeping, chronic headaches, memory loss, mood disorders, uveitis, and other visual and auditory problems^3–7^. Studies place the overall prevalence of PEVDS at over 75%, with the cumulative incidence of symptoms rising until 2 years post-discharge^4,5^. Many components of PEVDS are painful, interfere with daily functions and result in decreased quality of life^3,5^.

Despite the severity of the issues facing EBOV survivors, the origin and nature of PEVDS neurological manifestations are still not entirely understood. Extracranially, EBOV is known to infect endothelial cells and trigger pro-inflammatory cytokine release^8^. It is unclear whether a similar process occurs in the brain, resulting in a compromised blood–brain barrier (BBB). EBOV has already been identified in the cerebrospinal fluid (CSF) of several patients in both the acute and late-onset stages, suggesting that the virus is able to bypass the BBB^9–11^. Although recently a replication-competent pseudotyped vesicular stomatitis virus (VSV) platform where the VSV GP was replaced with Ebola’s GP (rVSVΔG-ZEBOV-GP) showed infection of retinal, brainstem and cerebellar neurons in neonatal mice^12^, there has been no documentation of neuronal or microglial infection with the non-pseudotyped virus in natural hosts, ex vivo.

Some studies suggest that the molecular and functional damage caused by EBOV within the central nervous system (CNS) improves over time^13,14^. However, without pre-disease baseline assessment of those patients, the extent of recovery is difficult to determine. Studies of EBOV infection in humans remain complicated by the urgency and resource-limited settings of the outbreaks. Animal models of EBOV, therefore, are a pathway to understand the neurological implications of the virus infection in the CNS during the acute stage of illness. Nonhuman primate (NHP) models of EBOV infection are well-described^15^. In these models, as in infected patients, EBOV infects macrophages, monocytes and dendritic cells, which eventually seeds multiple organs, including the liver, spleen, eyes and lungs, typically leading to death within 6 to 8 days of inoculation^15^.

Molecular imaging of EBOV infection offers the ability to non-invasively and longitudinally monitor various features of the disease including CNS involvement. Furthermore, imaging studies can shed light on the pathophysiology of lingering neurological abnormalities in survivors. In this study, we employed magnetic resonance imaging (MRI) and ^18^F-Fluorodeoxyglucose positron emission tomography/computed tomography (FDG PET/CT) to track changes in brain anatomy, BBB integrity and brain glucose metabolism in EBOV-infected macaques. We serially measured biomarkers of disease in the blood and in one group of animals, in the CSF. We correlated the laboratory findings to imaging. Finally, we used multiplex fluorescence immunohistology to screen for relevant biomarkers of neuroinflammation and neurodegeneration in brain tissues post-necropsy.

In this work, using a combination of MRI (quantitative MR-relaxometry) and FDG PET/CT, we show brain involvement with EBOV in acutely infected macaques, including BBB dysfunction and metabolic changes, which correlate with disease progression and are confirmed with immunohistopathology.

## Results

### Disease progression biomarkers

**Fig. 1:**
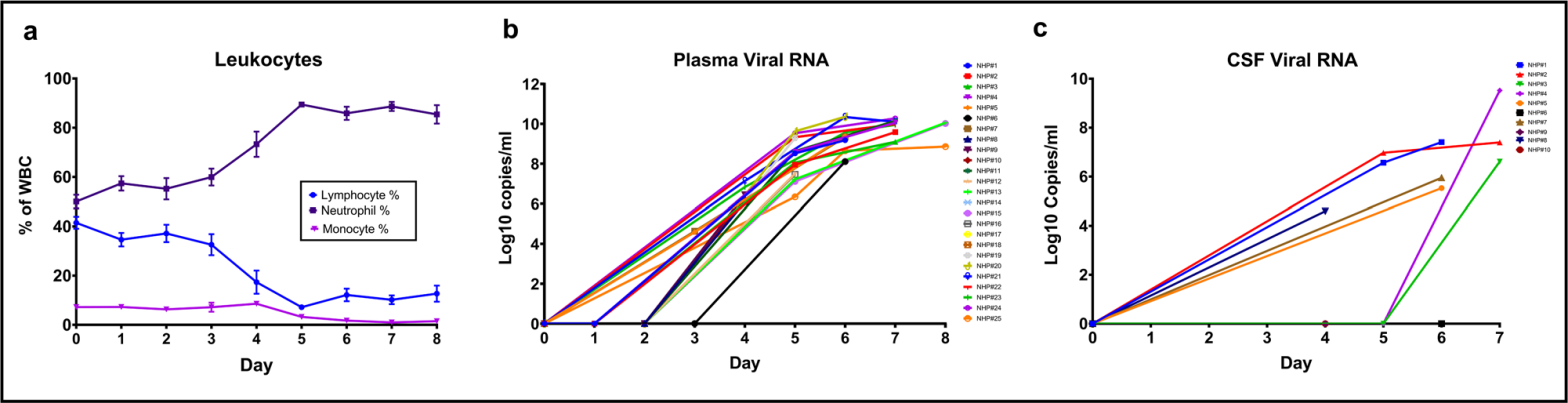
Complete blood counts, plasma and CSF viral loads in infected animals. **a** Percent lymphocytes, neutrophils and monocytes in the blood of all animals (*n* = 25 animals) over the course of the disease. **b** Plasma viral load values for all animals (*n* = 25 animals) over the course of the disease. **c** CSF viral load values for group A animals (*n* = 10 animals) over the course of the disease. Data are presented as mean values +/− standard error of the mean (SEM). Source data are provided as a Source Data file.

We evaluated our animals for progression of the systemic infection using complete blood counts with differential at different time points after inoculation, in order to compare to human disease progression, as well as correlate with our imaging findings. We expected the infected animals to develop progressive lymphopenia, monocytopenia, and decreased platelet counts, as has been described in this animal model before^15^ and in infected patients^16–18^. At days 4 and 5 post-infection, platelets, lymphocytes, reticulocytes, and monocyte counts and percentages all began to gradually decrease, while neutrophil counts/percentages increased (Supplementary Table 1 and Fig. 1a). Those results were expected and to a large degree paralleled similar disease progression markers in patients, especially in fatal cases^19,20^, likely reflecting direct infection of monocytes/macrophages and bystander lymphocytic apoptosis^16–18^.

In following up of viremia, there was no viral RNA detected in the plasma of group A and group B animals on day 2, while on day 3 viral RNA loads were measurable in almost all assayed animals and increased thereafter (Supplementary Table 1 and Fig. 1b). In group A that underwent CSF sampling, on the other hand, viral RNA was detected in the CSF by days 4 and 5 post-infection with dramatic increases seen in some animals by day 7 (Supplementary Table 1 and Fig. 1c). This was similar to described cases of positive EBOV RNA identified in the CSF of multiple infected patients, consistent with CNS involvement and breach of the BBB^9,11,21^.

We measured plasma cytokine levels in our animals to document and monitor the development of the cytokine storm described in patients with EBOV infection^22^, especially fatal cases. Plasma cytokines levels, reflecting both pro-inflammatory as well as anti-inflammatory systemic responses to the infection, increased gradually, with interleukin (IL)-10 and IL-6 beginning to increase on day 4. IL-8 and tumor necrosis factor alpha (TNF-α), on the other hand, began increasing on day 6, while GM-CSF and IL-2 began increasing on day 7 (Supplementary Table 2 and Supplementary Fig. 1). The cytokine changes in the animals are similar to those described in EBOV-infected patients, especially concerning the increases in pro-inflammatory cytokines such as IL-6 and IL-8^23,24^, as well as the anti-inflammatory cytokine IL-10^23^. In group B animals, the increases of cytokine levels over time were statistically significant for IL-2, IL-6, IL-8, and IL-10 (linear mixed models (LMM), *p* < 0.01).

Unlike in the plasma, changes of cytokine levels in the CSF of acutely infected EBOV patients have not been described, likely due to the difficulty of obtaining and analyzing CSF in underdeveloped countries with limited resources. In this study, we longitudinally evaluated changes in CSF cytokines in the infected animals to better understand the pathophysiology of CNS involvement following infection and to correlate those results to our imaging findings. In the CSF, levels of MCP-1, IL-1Ra, IL-2, G-CSF, and IL-18 started increasing on day 5 post-infection. Increases in IL-15, IL-1β, IL-6, and TNF-α levels were slightly more delayed in the CSF, beginning closer to day 6 post-infection. Levels of IFN-γ were not appreciably elevated. Levels of IL-10, TGF-α, IL-13, IL-4 and MIP-1α did not increase appreciably in the CSF (Supplementary Table 3). The increases in CSF cytokine levels over time were statistically significant for MCP-1, IL-1Ra, IL-6 and IL-8 (LMM, *p* < 0.01) (Supplementary Fig. 2). Similar increases in CSF cytokines have been previously reported in patients with other viral meningitides^25–27^.

Finally, we found viable virus in various brain tissues from group A animals, as determined by plaque assay, mainly on days 6 and 7 after inoculation. The distribution was not very different across regions (Supplementary Fig. 3). This could however be partially due to the lack of perfusion of the animals at necropsy and tissue collection, with secondary confounding effect of high blood viremia.

### Findings from MRI

Measuring MR relaxation times (e.g., T1 and T2 values) has long been used to evaluate developmental and pathologic changes in the brain at the molecular level^28–30^. In MR physics terminology, T1 relaxation (also known as longitudinal relaxation) is the process by which the net longitudinal magnetization of a certain tissue returns to baseline value after being subjected to a radiofrequency pulse. T1 value is defined as the time required for the longitudinal magnetization value to grow to (1−1/*e*) or ~63% of its original value^31^. T1 values depend on tissue composition with bound hydrogen atoms such as those within macromolecules or in the vicinity of large macromolecules having short T1 values while unbound hydrogen atoms such as in water (e.g., CSF) have very high T1 values^31^. T1 values of various tissues, thus, reflect the consistency of those tissues^32,33^ and fluid accumulation in the extracellular space is expected to increase those values compared to baseline^30,34,35^, mainly because the CSF T1 value at 3 T (~4000^32^) is much higher than that of white and gray matter (806 ± 25 and 1460 ± 114, respectively, at baseline). Contrast extravasation into the extracellular space due to BBB disruption, on the other hand, would result in further shortening of the T1 values due to the inherent T1-shortening effects of Gadolinium-based contrast materials through their paramagnetic properties^36^.

In order to control for factors that could influence measured brain T1 values, we performed multiple experiments to assess the accuracy and precision of those measures, using gel phantoms and control animals. We found that the dual-flip-angle method measures of T1 relaxation times differed <2% compared to the actual inversion recovery measures of the gel phantoms, a level of accuracy that is comparable to the one reported by Eminian and colleagues^29^. When the phantoms were scanned at different times, days, and orientations, the coefficient of variance remained around or below 4% in the range typical for white and gray matter. Experiments with NHPs scanned multiple times within one day demonstrated a coefficient of variation of T1 values within 10 selected volumes of interest (VOIs) in the brain of <3%.

Another quality assurance issue in group A animals was the variability in age and size of the animals, which could potentially affect co-registration of the individual scans to the D99 atlas, which we used to delineate the VOIs. In order to avoid mis-registration that could affect the final T1 values, we carefully inspected the quality of co-registration between the MR scans and the D99 atlas in all animals. We found the quality of co-registration to be appropriate irrespective of the animals’ ages and weights (Supplementary Fig. 4).

**Fig. 2:**
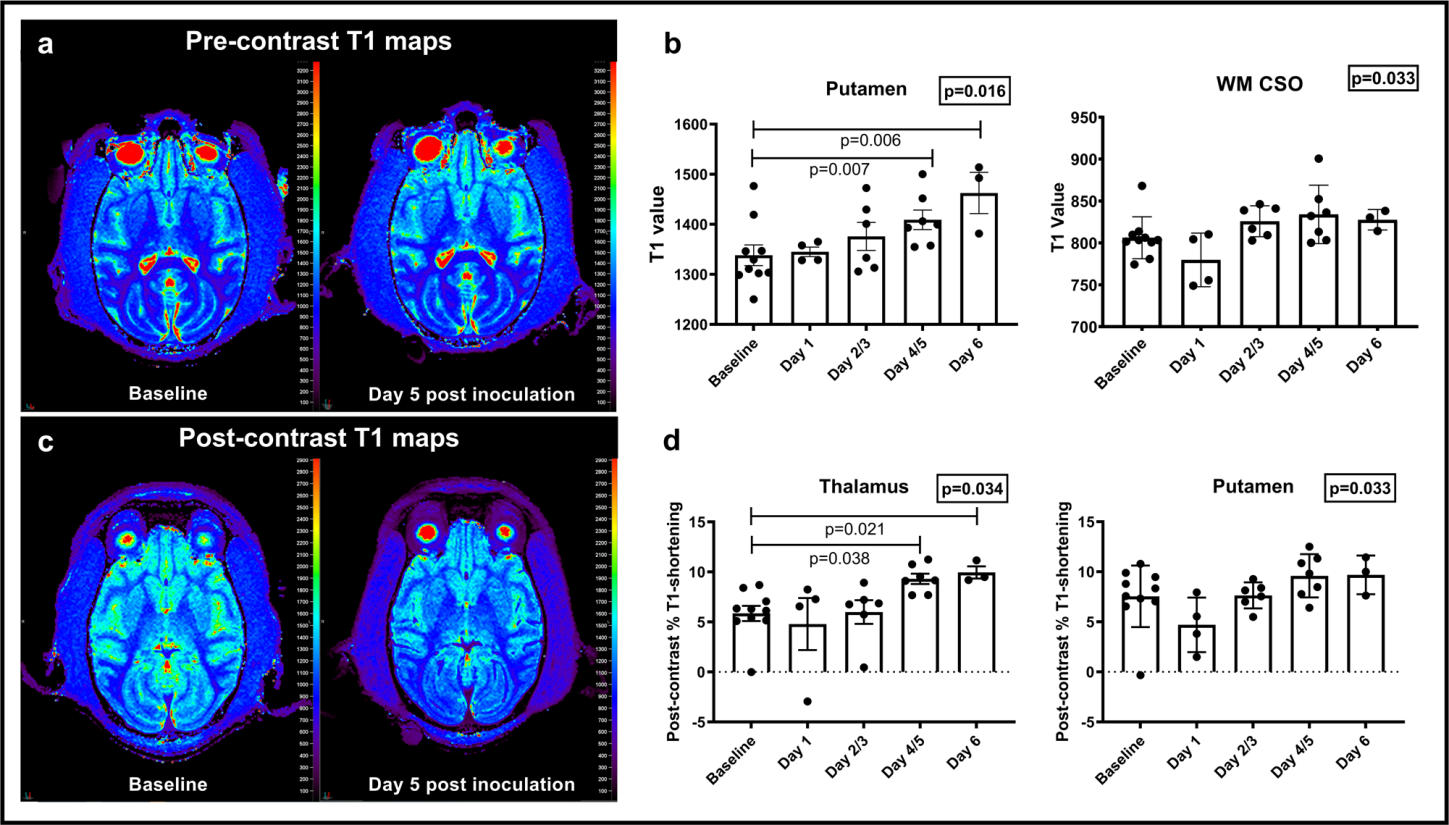
Longitudinal T1-relaxometry changes in infected monkeys. **a** Qualitative T1 maps at baseline and on day 5 after inoculation in one representative animal show subtle increased T1 values especially in the basal ganglia. **b** Increases in T1 pre-contrast values are noted in the putamen and white matter centrum semiovale over the course of the disease (*n* = 10 animals, Linear mixed effect model, S-plus 8.2). Data are presented as mean values + /− SEM. **c** Qualitative post contrast T1 maps in one representative animal show further decreased T1 values on day 5 after inoculation compared to baseline. **d** Increases in Post-contrast % T1-shortening in the putamen and thalamus over the course of disease (*n* = 10 animals, Linear mixed effect model, S-plus 8.2). Data are presented as mean values + /− SEM. Source data are provided as a Source Data file.

In the infected animal brains, we found that time post-infection was associated with increases in pre-contrast T1 values in most of the evaluated regions, that in some animals could be visually detected on T1 maps (Fig. 2a), with a trend for significance seen in the centrum semiovale and putamen (LMM, *p* = 0.033 and 0.016, respectively) (Fig. 2b). In the putamen, the increases were most noticeable between days 4/5 and baseline and between day 6 and baseline (LMM, *p* = 0.007 and 0.006, respectively). Our findings supported increased water content in those regions over time, suggesting disruption of the BBB and secondary mild brain edema due to accumulation of fluid in the extracellular spaces^30,34^.

We also found further drop in T1 values (increases in %T1-shortening) after contrast administration in most regions, that in some animals could be visually detected on post contrast T1 maps (Fig. 2c). Time post-infection showed borderline significant association with %T1-shortening in the thalamus and putamen (LMM, *p* = 0.034 and 0.033, respectively) (Fig. 2d). Those findings suggested extravasation of Gadolinium-based contrast materials into the extracellular space in the brains of infected animals, over time, due to BBB disruption^36^.

### Correlations between MRI data and biomarkers

Positive correlations were identified between increasing plasma viral load and increasing T1 values in the caudate and putamen (*p* < 0.01) (Supplementary Table 4a). We also found positive correlations between plasma viral load and %T1-shortening in the putamen, thalamus and cerebellar gray matter (LMM, *p* < 0.01). CSF viral load did not correlate with T1 values in any region however it correlated positively with %T1-shortening in cerebellar gray matter and frontal cortex (LMM, *p* < 0.01) (Supplementary Table 4b).

The changes in MR measures suggestive of BBB disruption also correlated with different inflammatory markers in the plasma and CSF. We found positive correlations between frontal cortex T1 values and CSF levels of MIP-1α and GM-CSF (LMM, *p* < 0.01) (Supplementary Table 4a), as well as between %T1 shortening and CSF levels of IL-1Ra, IL-1β, IL-8, IL-18, GM-CSF, MIP-1α and MIP-1β (LMM, *p* < 0.01) (Supplementary Table 4b). Our results supported a relationship between neuroinflammatory changes and the degree of BBB dysfunction associated with EBOV infection.

### Findings from FDG PET/CT

**Fig. 3:**
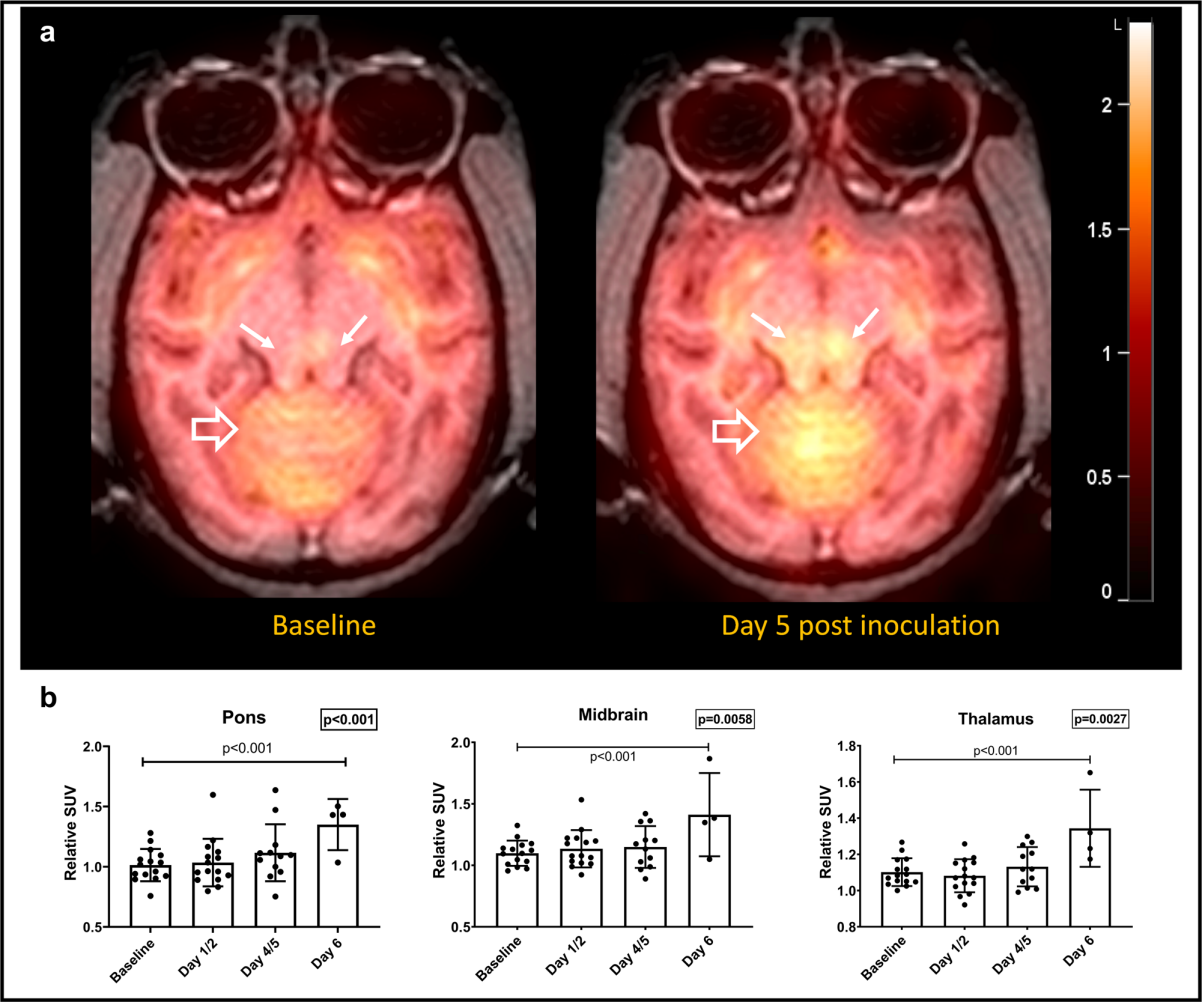
Longitudinal FDG PET changes in infected monkeys. **a** Parametric relative SUV maps from a representative EBOV-infected animal showing increased uptake in the brainstem (solid white arrows) and cerebellum (open white arrows) on Day 5 post inoculation compared to baseline. **b** Significant increases in relative FDG uptake are seen in the thalamus, midbrain and pons over the course of disease (*n* = 15 animals, Linear mixed effect model, S-plus 8.2, *p* = 0.0027, 0.0059 and 0.0007, respectively). Data are presented as mean values + /− SEM. Source data are provided as a Source Data file.

FDG is a non-physiological glucose analog that undergoes similar cellular uptake as glucose by glucose transporters (GLUTs) such as GLUT1 and GLUT3. Once in the cell, FDG undergoes phosphorylation by the enzyme hexokinase. Unlike glucose, however, the phosphorylated FDG molecule does not undergo glycolysis and instead gets entrapped in the cell. As such, FDG PET reflects the extent and distribution of glucose metabolism in various tissues^37^. Since glucose is the only source of energy for neurons, there is usually high FDG uptake in cortical and subcortical areas of the brain. Neuroinflammation, however, can result in increased FDG uptake mainly due to increased uptake by activated immune cells^38^. In this study, we used FDG PET to assess changes in the extent and distribution of brain glucose metabolism between the baseline (pre-inoculation) and various time points after inoculation. We evaluated both the absolute mean standardized uptake values (SUVs) in different regions of the brain as well as the relative SUVs, which are normalized to whole brain uptake. The latter approach is commonly used in the literature and is known to provide a more stable quantitative assessment of cerebral glucose metabolism than absolute SUV values^39^. We did not find statistically significant changes for whole brain FDG uptake over time (LMM, *p* > 0.01). Regional absolute SUVmean values appeared to increase as a function of time in multiple brain regions however those changes were borderline significant only in the pons (LMM, *p* = 0.015). Relative SUVs in the thalamus, midbrain and pons, on the other hand, exhibited significant overall increases over time (LMM, *p* = 0.003, *p* = 0.006, *p* = 0.0007) (Fig. 3a, b). Relative SUVs in these regions increased significantly at day 6 compared to baseline (LMM, *p* < 0.001). Borderline significant increases in relative SUV values were seen in the medulla and caudate (LMM, *p* = 0.02 and 0.04). Using linear mixed effect regression models, correlations were identified between regional relative SUV values and biological markers of disease. Relative SUVs of the pons positively correlated with blood viremia, as well as plasma levels of IL-8, IL-6, and IL-10 (LMM, *p* < 0.01). Relative uptake in the thalamus, midbrain and medulla correlated significantly with IL-8 and IL-6 (LMM, *p* < 0.01) (Supplementary Table 4c).

### Findings from structural MR imaging

Structural MR imaging is essential for the detection of macroscopic abnormalities in the setting of infection. Those could include abnormal high signal intensity on FLAIR and T2 weighted images, microhemorrhagic changes, best seen on susceptibility-weighted imaging, and abnormal parenchymal and/or meningeal enhancement after contrast administration. In our animals, however, MPRAGE and pre/post contrast T1-weighted (Flip angle 32^o^) images scans on group A and B animals, as well as PD/T2 weighted and pre and post FLAIR images on group C animals showed no evidence of abnormal signal intensity, distorted anatomy or abnormal meningeal or parenchymal enhancement (Supplementary Fig. 5). On T2* weighted images, venous congestion was identified in the deep and superficial venous systems in the later stages of the disease, with however no evidence of intraparenchymal acute hemorrhage, microhemorrhagic changes or hemosiderin deposition. Evaluation of the orbits, including globes and retro-orbital tissues, showed no visually detectable changes between baseline and post inoculation scans (Supplementary Fig. 5). In group B animals that did not get an MRI scan of the brain, diagnostic quality brain CT scans obtained from the FDG PET/CT examinations did not show any gross abnormalities.

### Immunohistochemistry and immunofluorescence

**Fig. 4:**
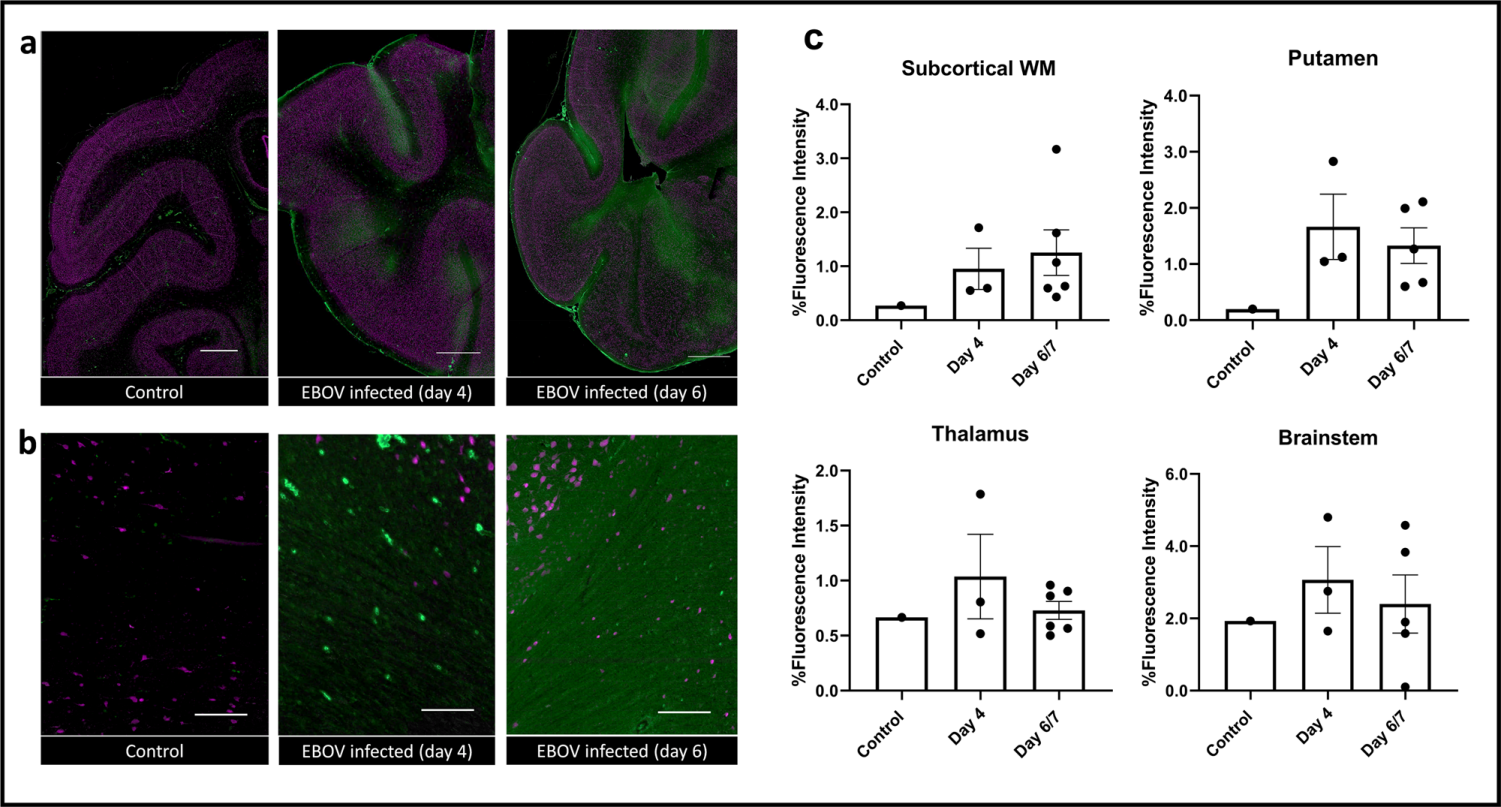
Multiplex fluorescence immunohistochemistry showing extravascular albumin leakage in the infected animal brains. **a** Low-magnification images show patchy increased staining for albumin (green) mainly in the subcortical white matter of infected animals compared to a control animal, increasing on day 6 compared to day 4 post inoculation. Neurons are stained in purple (NeuN). Scale bar = 2 mm, magnification 3%. **b** Extravascular staining for albumin in infected animals is better seen on higher magnification images of the gray matter/white matter junction. Scale bar = 150 µm, magnification 50%. **c** Quantification of Albumin immunofluorescent staining in the subcortical white matter, putamen, thalamus and brainstem show increased percent fluorescence intensity in the infected animals compared to control (*n* = 10 animals). Data are presented as mean values + /− SEM. Source data are provided as a Source Data file.

In order to better understand the pathophysiological bases underlying the imaging findings in our animals, we performed elaborate immunofluorescent staining of various cell populations in the brains of euthanized animals, at different time points after inoculation. We also stained for the presence of extravascular albumin as a measure of increased BBB permeability. In the control animal, albumin staining was seen solely within the vessels, as expected, since albumin and many other proteins are largely excluded by a functioning BBB^40^. In the infected animals, on the other hand, we identified extravascular albumin staining, which increased between day 4 and day 6/7, both qualitatively (Fig. 4a, b) and quantitatively (Fig. 4c). Extravascular albumin staining indicates prior BBB disruption, allowing protein extravasation into the extracellular spaces of the brain. Interestingly, the pattern of albumin extravasation was regional and somewhat patchy, mainly identified in the striatum and subcortical white matter.

**Fig. 5:**
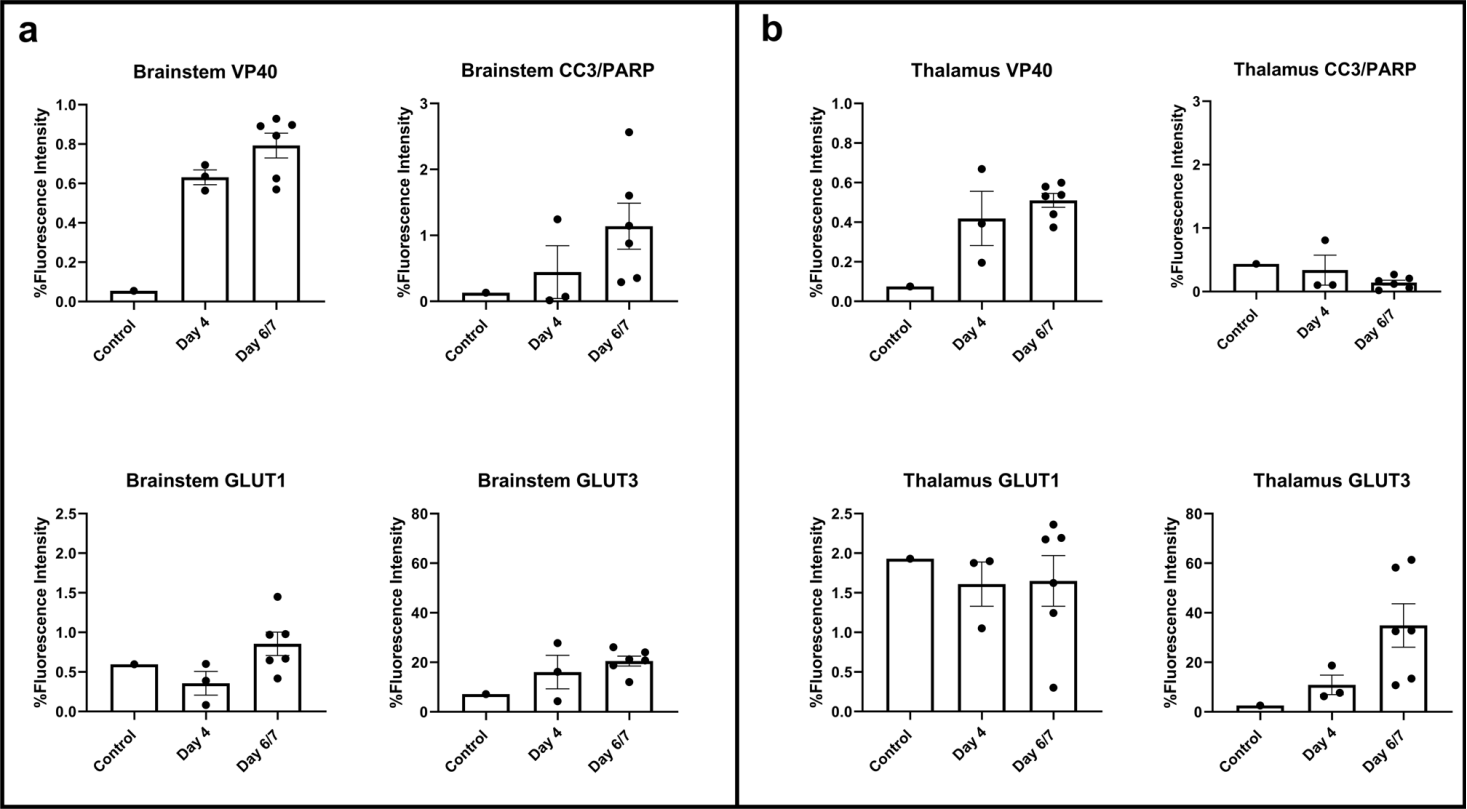
Quantification of multiplex fluorescence immunohistochemistry staining for viral antigen (VP40), apoptosis (CC3/PARP1) and glucose transporters (GLUT1 and GLUT3). **a** There is increased VP40, CC3/PARP1, GLUT1 and GLUT3 staining in the brainstem, more so on days 6 and 7 compared to day 4 post inoculation (*n* = 10 animals). **b** There is increased VP40 and GLUT3 staining in the thalamus of infected animals, more so on days 6 and 7 compared to day 4 post inoculation (*n* = 10 animals). Data are presented as mean values + /− SEM. Source data are provided as a Source Data file.

**Fig. 6:**
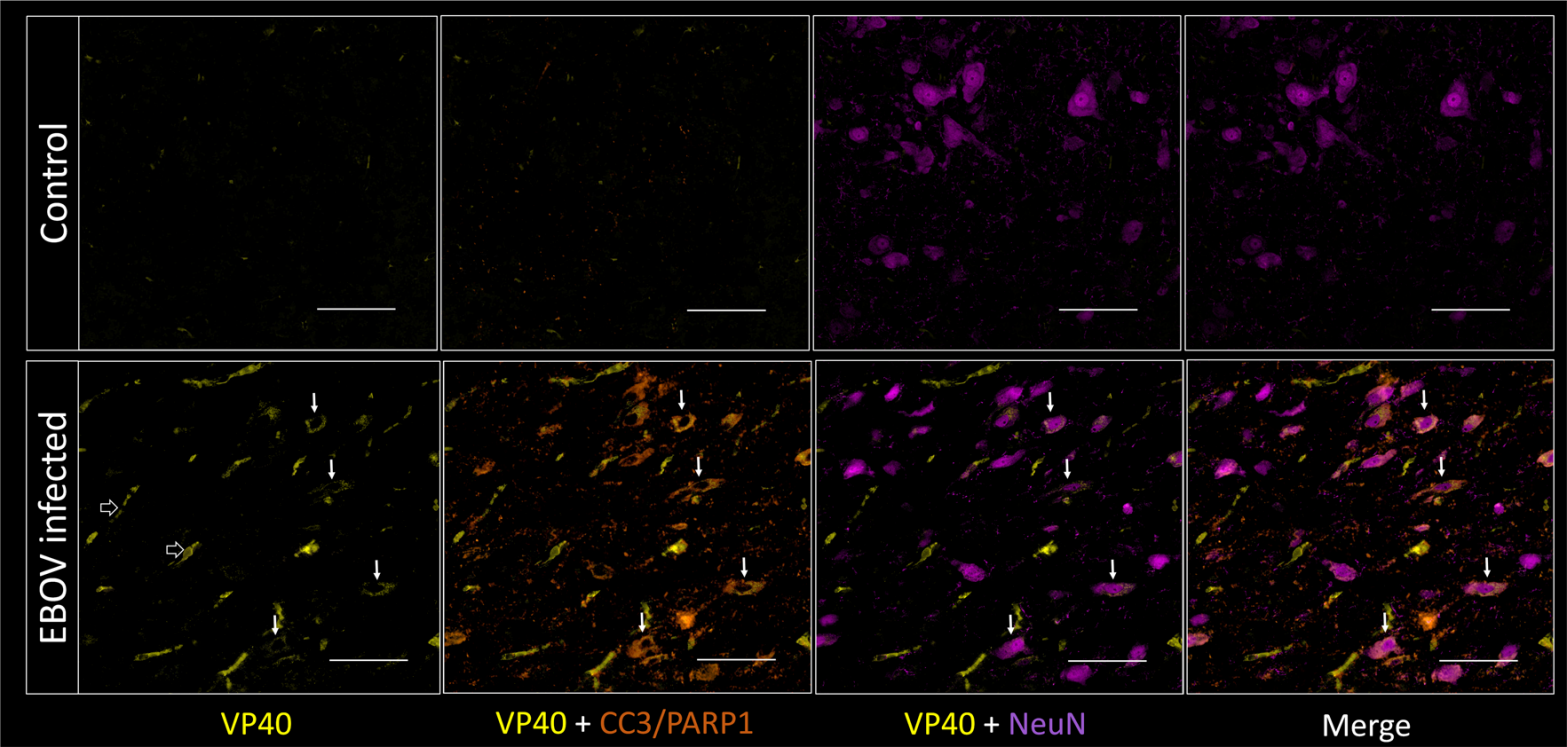
Multiplex fluorescence immunohistochemistry staining for viral antigen (VP40; yellow), apoptosis (CC3/PARP1; orange) and neurons (NeuN; purple) in the brainstem of control and infected animals. VP40 staining is seen in the vessels of the infected animal (not perfused) (white open arrows). VP40 is also overlapping with neuronal and apoptosis markers (small solid white arrows), suggesting infection and injury of multiple neurons in the field of view. Staining was performed for nine infected and one control animal. Scale bar = 100 µm, magnification 100%.

We also stained the brains of our animals for the presence of the EBOV antigen, VP40, to assess for infected cell populations, and for cellular apoptosis using CC3/PARP1 staining^41^. Quantitatively, higher staining for VP40 (EBOV antigen) was noted in the infected animals compared to the control animal, in the thalamus and in the midbrain, increasing further on day 6/7 compared to Day 4. Increased CC3/PARP1 staining, reflecting cellular apoptosis, was also noted, mainly in the brainstem (Fig. 5a, b). VP40 staining co-localized with endothelial cells (CD31/CD144) (Supplementary Fig. 6), as expected, since endothelial cell infection is a known manifestation of the infection^8,42,43^. VP40 staining also co-localized with circulating cells in the vascular lumina consistent with infected blood cells, mainly monocytes, which was expected since the animals were not perfused during euthanasia. Some VP40 staining co-localized with microglia/macrophages in the brain parenchyma, suggesting infection, which is not surprising since monocytes and macrophages are known to be susceptible to infection by EBOV^15^. The majority of the cells staining for VP40, however, also stained for NeuN, a neuronal marker, suggesting neuronal infection (Fig. 6). Apoptosis, as demonstrated by staining for CC3/PARP1, was observed mostly in the brainstem and often co-localized with NeuN and VP40 staining (Fig. 6). Those findings suggested that some neurons, especially in the brainstem, are susceptible to EBOV infection, with secondary neuronal injury and death. There were however multiple apoptotic neurons (NeuN and CC3/PARP1 positive) that did not show co-staining with VP40, suggesting a distinct cause of neurotoxicity. This could be due to BBB disruption and secondary influx of neurotoxins into the brain parenchyma. One such neurotoxin is albumin, which has been shown to cause neuronal injury in the setting of disrupted BBB^44^. Further supporting this theory is that multiple neurons (NeuN-positive cells) appeared to stain for albumin as well.

We also stained the brains of our animals for the expression levels of glucose transporters (GLUT1 and GLUT3)^45^ in order to better understand the FDG PET findings of increased regional metabolism in the infected animals compared to baseline. The expression of GLUT1, a glucose transporter, which in the brain is mainly expressed in endothelial cells and astrocytes^46^, was mainly increased in the brainstem (Fig. 5a). At the same time, the expression of GLUT3, a neuronal glucose transporter^46^, was seen both in the thalamus and in the midbrain, increasing further on day 6/7 compared to Day 4 (Fig. 5a, b). Staining for GLUT3 was especially noted in apoptotic neurons, compared to non-apoptotic neurons (Supplementary Fig. 7), suggesting increased metabolic demands of the former.

Finally, Iba1 staining showed evidence of thickening and retraction of the microglial processes compared to normal (Supplementary Fig. 8a). Quantitatively, Iba1 staining quantification was increased in the brainstem and decreased in the thalamus. Although we did not see gross ameboid morphology of microglia/macrophages, we did however identify an increase in the size of the microglial cell bodies in the infected animals, which became more evident on terminal day compared to day 4 (Supplementary Fig. 8b). This was associated with shortening and thickening of the microglial processes. Our findings suggest early rather than late neuroinflammation at which point we would have seen a more reactive phagocytic microglial phenotype^47^.

## Discussion

The extent of EBOV-induced neurological disease was not well elucidated during the 2016 epidemic in West Africa, or recent 2018 epidemic in the DRC, due to the high mortality associated with the infection and lack of local infrastructure allowing for sophisticated imaging, with only a few papers showing sporadic cases of patients with abnormal imaging findings^10,14^. The prevalence of neurological sequelae in EBOV survivors^4,5^, however, has reignited the interest in CNS involvement with EBOV. Using a combination of MR imaging including T1-relaxometry and FDG PET/CT imaging, which was further expanded to higher spatial resolution at the cellular level using MF-IHC staining and imaging, we identified BBB disruption, mild neuroinflammation, as well as sporadic neuronal VP40 antigen positivity (infection) and apoptosis, as the main markers of early CNS involvement in an animal model of EBOV disease. Our findings have not been previously described with EBOV, to our knowledge, and could potentially explain some of the CNS manifestations of EBOV infection, both in the acute stage and in survivors.

With T1 mapping, we detected an increase in T1 pre-contrast values as well as %T1-shortening over the course of the experiment, as disease severity intensified (Fig. 2). Altogether, these MR findings are suggestive of BBB disruption, secondary fluid accumulation and contrast extravasation into the extracellular space^30,34,35^. Our imaging results were corroborated by extravascular albumin staining in infected animals (Fig. 4), especially later in the disease process. We hypothesize that the disrupted BBB is likely related to increased peripheral cytokine levels known to induce BBB disruption, especially IL-6^48–50^, as well as endothelial cell infection (shown as VP40 co-staining of endothelial cells throughout the brain vasculature, Supplementary Fig. 6). In further support of this hypothesis, MR measures of T1 and %T1-shortening values significantly correlated with other indicators of disease progression: caudate/putamen T1 values and putamen/thalamus %T1-shortening correlated positively with plasma viral load (*p* < 0.01), suggesting an ongoing process that overlaps with peripheral disease progression and CNS involvement. The correlations with other measures of inflammation in the CSF (Supplementary Table 4) suggest an additional ongoing inflammatory process, possibly further contributing to BBB dysfunction^51^.

Further insight into the effects of EBOV on the CNS was provided by FDG PET/CT imaging in a separate group of animals. In that cohort, EBOV infection was associated with incremental regional increased FDG uptake, especially in the brainstem (pons and midbrain) and thalamus, over the disease process. Relative uptake values also correlated with peripheral measures of the disease, such as plasma viral load and plasma cytokines (IL-8, IL-6, and IL-10) (Supplementary Table 4). On the cellular level, using MF-IHC, we detected mild early microglial activation (Supplementary Fig. 8). However, we also identified multiple neurons co-staining for NeuN (neuronal marker) and VP40 (EBOV viral protein). Many of those stained positively for CC3/PARP1, consistent with apoptosis, starting on day 4 post inoculation, and progressing over time (Figs. 5 and 6). More interestingly, we identified an appreciable increase in GLUT3 expression, which also progressed over time, especially in association with apoptotic neurons (Supplementary Fig. 7). There was also mild increase in GLUT1 expression in the brainstem on Days 6/7. Our findings are suggestive of sporadic neuronal infection, injury and apoptosis, with neuroinflammation and associated increased metabolic demands.

The mechanism of neuronal involvement with EBOV can be explained by the known expression of the Niemann-Pick-C1 (NPC-1) receptors in neurons^52,53^. It has been already demonstrated that the NPC-1 protein is essential for viral entry, independent of the cholesterol transport function of the receptor^54^. This makes NPC-1 a target for antiviral therapy based on preventing the viral glycoprotein from binding to it^54,55^. Interestingly, although NPC-1 is expressed in the majority of neurons throughout the brain, significantly higher levels of expression were previously described in the rodent cerebellum and pontine nuclei^53^, with the latter showing more abnormalities than the rest of the brain in our imaging studies.

Our study used a combination of MRI, PET imaging and immunohistology to better understand CNS involvement in the acute disease phase following EBOV infection. EBOV-infected subjects often develop a picture of sepsis with multi-organ failure near the end of the disease process, resulting in high fatality. The contribution of sepsis and septic shock to neurologic damage in EBOV-infected patients cannot be ruled out considering that patients who have experienced severe sepsis from other etiologies often demonstrate deterioration in cognitive function, lasting for years after the event, when compared to other hospitalized patients who did not suffer from sepsis^56^. We did, however, attempt to minimize the effect of sepsis and multi-organ failure on our quantitative analysis of the imaging studies by eliminating the last two days of the disease, which are characterized by significant deterioration and shock in this model. Also, our findings of endothelial and neuronal involvement with the virus, prior to establishment of sepsis and multi-organ failure in the animals, indicate an important role for EBOV that goes beyond the confounding effects of sepsis and warrants more careful investigation.

Neurological deficits and mood disorders are relatively common components of PEVDS that range in severity and can last for months after recovery and viral clearance^7^. Studies in human survivors have found or suspected meningoencephalitis at multiple time points, anywhere from several weeks to multiple months post-infection^10,11^. EBOV RNA was identified in the CSF of multiple infected patients^9,11,21^. Similarly, this has been also observed with other viral hemorrhagic fever infections, such as Lassa virus^57^ and various arbovirus infection such as Zika, Chikungunya and Dengue viruses, where patients presented with neurologic problems^57^. The question of neuronal infection with EBOV, however, has not been answered. A recent study using a replication-competent pseudotyped vesicular stomatitis virus (VSV) platform where the VSV GP was replaced with Ebola’s GP (rVSVΔG-ZEBOV-GP) showed infection of retinal, brainstem and cerebellar neurons in neonatal mice^12^. Another recent preliminary report also showed the ability of EBOV to infect neuronal cells in vitro, with productive or restrictive replication within various neuroblastoma cell line types^58^. To our knowledge, however, there has been no previous ex vivo demonstration of direct neuronal infection with the non-pseudotyped EBOV.

Our findings of focal hypermetabolism on FDG PET/CT pointed us towards assessment of neuroinflammation and expression of glucose transporters. Although there was slightly increased Iba1 expression histologically with increased cell body size and shortening of the processes consistent with neuroinflammation (Supplementary Fig. 8), we felt there must be another mechanism occurring, especially in the brainstem, to account for increased glucose metabolism. An overexpression of neuron-specific GLUT3^46^ coinciding with neuronal apoptosis, suggested this could be an additional mechanism of increased FDG uptake in our animals. GLUT3 upregulation has been previously described with other models of brain pathology such as lead exposure^59^, hypoxia^60^, and severe traumatic injury^45^. The exact pathophysiology of increased GLUT3 expression in our infected monkey model combined with neuronal apoptosis could be due to glutamate excitotoxicity^61^ with similar increased expression seen in association with impending apoptosis in hypoxic brain injury^62^. Interestingly, not all of the apoptotic neurons in our infected animals were positive for VP40 indicating multi-factorial causes for neuronal injury. One possibility includes neuronal absorption of extravasated albumin, which has been demonstrated to cause neuronal injury in the setting of disrupted BBB^44^. In fact, in our animals, we noticed that many neurons stained positively for albumin, especially ones that were located in close vicinity to extravascular albumin accumulation. Many of those neurons appeared to be apoptotic.

Our abnormal imaging findings on MRI and FDG PET/CT increased over time. This was paralleled by increased number of VP40-positive neurons, apoptosis and albumin extravasation seen on days 6–7 after inoculation compared to day 4 in the same brain regions (thalamus and brainstem). Based on those results, it is possible that the degree of pathology seen in patients with PEVDS would depend on the degree and duration of acute infection in those who ultimately survive the disease. This is supported by positive correlations between imaging findings and peripheral measures of disease including plasma viral load and cytokine levels. The neurological symptomatology in acute EBOV includes headaches with reports of meningitis and encephalitis^3,6,7^. Headache is a non-specific symptom but could possibly reflect subtle edema due to BBB disruption consistent with our imaging findings, and/or meningeal inflammation supported by increased CSF cytokines. Neurological sequelae in survivors are also vague and non-focal, including headaches, short-term memory issues, dizziness and paresthesias. Interestingly, however, objective neurological examination in a group of survivors showed impairments of either pursuits or saccades in nearly two-thirds of the cohort^7^. Those deficits usually indicate brainstem dysfunction namely in the midbrain (vertical saccades) and pons/medulla (horizontal saccades)^63^, and/or in the subcortical structures. All those regions, especially the brainstem, have shown abnormal T1 values and/or increased FDG uptake in our animals. We also found most of the infected apoptotic neurons in the brainstem. As such, we believe our findings are consistent with symptomatology in survivors. More work needs to be done, however, to support our hypotheses, and the large population of survivors offers both the opportunity and the rationale to continue these inquiries.

The pathologic changes in our EBOV-infected NHPs were mostly noted in the brainstem, thalamus and basal ganglia. This could be in part related to higher expression of the NPC-1 receptor in those regions^53^. A predilection for certain areas in the brain has been described with multiple other viral infections, including CMV involving the ventricular walls, HIV involving the basal ganglia, HSV involving the frontal and temporal lobes and West Nile virus involving mainly the basal ganglia, thalamus, upper brainstem, and cerebellum as well as the anterior horns of the spinal cord^64–66^. More recently a similar predilection has been proposed for SARS-CoV-2: an autopsy series of COVID-19-infected patients showed involvement of the pons with evidence of pan-encephalitis, meningitis, and brainstem neuronal cell damage^67^. Whether the pattern of involvement we describe here for EBOV is specific or commonly seen in other filovirus infections remains to be determined.

Our study has limitations. The use of the acute and fatal EBOV-infected NHP model limits the generalizability of our findings to patients who received successful care regimens, survived longer and still developed PEVDS. We also obtained CSF specimens in only one of our animal groups (group A, *n* = 10), however we were able to evaluate other markers of disease in group B, including peripheral viral load and plasma cytokines. Although we performed plaque assays to measure infectious virus in different brain regions in group A, the lack of perfusion of the animals at necropsy limited the usefulness of the data due to the confounding effect of high blood viremia. We did not perform partial volume correction on the PET scans, however we contracted the VOIs circumferentially to decrease the effect of spill in/spill out of radioactivity between the various regions (and to decrease the potential of partial averaging with adjacent CSF containing structures on MRI). In addition, considering our findings were all detected longitudinally using baseline imaging for comparison, with no appreciable anatomical changes over the observation period, we do not believe there was a major negative impact of partial volume effect on our measures of FDG uptake. Our animals could not be perfused prior to euthanasia, due to BSL-4 logistics, which probably affected the quantification of immunofluorescence. We had access to brain tissues from only one uninfected animal, which limits histological comparisons. An increase in staining of VP40, albumin, CC3/PARP1 and GLUT3 between day 4 and day 6/7 in the infected animals, however, confirms the observed pathology, which worsened over the course of the disease. Finally, we did not stain for neuronal expression of NPC-1 and instead relied on the published literature regarding expected distribution of NPC-1 expression in the brain^53^.

Moving forward, our imaging can be used to evaluate medical countermeasures in EBOV infection including various treatment methodologies, such as antiviral drugs and vaccines, for effectiveness in reducing/preventing CNS involvement in the wake of virus induced disease. EBOV outbreaks continue to occur, and any gains in knowledge can be harnessed to improve care for those who are acutely ill and to improve long-term outcomes for those who survive. Similar experimental paradigms can also be used to evaluate a variety of other infectious agents with proposed neurotropism, in appropriate animal models.

## Methods

### Animal care and use

All animal experiments were performed in a BSL-4 laboratory, were approved by the NIAID Division of Clinical Research Animal Care and Use Committee, and were performed in an AAALAC International accredited facility in accordance with relevant NIH policies and the Animal Welfare Act and Regulations.

For the MRI cohort (Group A), 10 male Indian origin rhesus macaques (*Macaca mulatta*, age range: 23–66 months, average age: 42.3 ± 19.9 months; average weight: 5.2 ± 1.8 kg) were used. All subjects were inoculated with 1000 plaque forming units of EBOV, Makona isolate (EBOV-Makona) via intramuscular injection. Two baseline brain MR scans were performed on each animal, and two or three MR scans were performed post-infection: on days 2 and 4 (*n* = 3), days 3 and 6 (*n* = 3), or days 1, 5, and 6/7 (*n* = 4).

For the FDG PET/CT cohort (Group B), 15 male Indian origin rhesus macaques (*Macaca mulatta*, age range: 31–91 months, average age: 48 ± 18.2 months, average weight: 5.6 ± 2.1 kg) were used. All subjects were inoculated with 1000 plaque forming units of EBOV-Makona via intramuscular injection. Group B was divided into a serial sacrifice subgroup (*n* = 7), in which animals were euthanized at day 2, day 5, and Terminal (days 7 or 8) post-exposure (*n* = 8). The terminal group subjects were allowed to progress until meeting clinical terminal endpoints^68^. All animals received two baseline FDG PET/CT scans, but the number of follow-up scans varied by subgroup assignment. Serial sacrifice animals euthanized at day 2 received one post-inoculation PET/CT scan immediately prior to euthanasia. Serial sacrifice animals that were euthanized at day 5 received two post-inoculation FDG PET/CT scans, at days 2 and 5, immediately prior to euthanasia. Terminal animals were scanned either 3 times (at days 1/2, 5, and 7/8) or 4 times prior to meeting endpoint criteria (at days 1, 4, 6, and 7/8).

A third group of male rhesus macaques (*n* = 2, *Macaca mulatta*, age range: 24-36 months, average age: 30 ± 8.5 months, average weight: 4.5 ± 0.5 kg), also infected with EBOV-Makona, were evaluated at baseline and on day 6 after inoculation using structural imaging including Proton-density/T2 (double-echo), pre and post contrast 2D T2 FLAIR images, as well as T2*-weighted imaging. The scans were evaluated qualitatively by a neuroradiologist (DAH) with 18 years of experience.

### Biomarker sampling

For all animals in the experiment, whole blood samples were obtained at least twice prior to EBOV exposure and subsequently on each post-infection scan day until euthanasia. Complete blood counts with differential (CBC/diff) were performed at different time points in all animals to assess disease progression. This included evaluation of white blood cell counts as well as counts and percentages of monocytes, lymphocytes, reticulocytes, neutrophils, and platelets. CBC/diff was determined from blood samples collected in ethylenediaminetetraacetic acid (EDTA)-coated blood tubes and analyzed using a Sysmex XT2000V™ (Sysmex America, Mundelein, IL). For Group A only, CSF samples were also acquired via cisternal puncture at baseline and subsequently on days 4, 5, 6, or 7 post infection.

Plasma and CSF qPCR assays were determined using the BEI Resources Critical Reagents Program (CRP) EZ1 quantitative reverse transcription PCR (qRT-PCR) kit. Serum and CSF cytokines were assessed using a Milliplex NHP primate kit (Millipore) that has been validated to cross react with rhesus macaques. Viral RNA from both plasma and CSF were analyzed with the same qPCR assay, which removed the inter-assay variability as a concern. In brief, viral RNA was extracted from 70 µl of sample that was inactivated by Trizol LS, and added to 280 µl of buffer AVL (Qiagen, Gemantown, MD Cat No. 19073). Samples were then extracted using the QIAamp Viral RNA Mini Kit (Qiagen, Germantown, MD Cat No 52904) in accordance with the manufacturer’s instructions, eluted in 70 µl of buffer AVE (Qiagen), aliquoted, and frozen. Five microliters of sample were assayed in duplicate reactions using the Ebola Zaire Target 1 LightCycler/Rapid Master Mix (Cat. No. BEI CRP PCR-EBZ-1R-K) and compared to a standard curve of serial tenfold dilutions (Cat No. BEI CRP Ebola Zaire Target 1 Custom Conc. Positive Control 1E + 9 copies/rxn). The lower limit of detection of our assay is 100 viral RNA copies.

For viral titration in different brain regions, tissues samples were first collected, snap frozen at -80^o^C and a 10% homogenate (w/v) was prepared in PBS using a Bead Ruptor Elite (Omni-inc., Georgia, USA). The tissue samples were then serially diluted and added to nearly confluent VeroE6 cells (BEI resources, a division of American Type Culture Collection (ATCC)) grown in multiwell plates. For the next hour, the virus adhered to and infected the cells (37 °C/5% CO_2_). Once the cells were infected, semi-solid 1.25% Avicel (FMC Biopolymer) diluted in EMEM was added to the corresponding wells, incubated for 5 days (37 °C/5% CO_2_) and eventually aspirated. At that point, the cells were fixed and stained using neutral buffered formalin (NBF, 0.4% crystal violet) for 30 min at room temperature. At the end of the assay, the plates were washed with water and the plaques were counted.

### Imaging procedures

All MRI scans, including those of Group A, and nine of the fifteen animals in Group B, were performed using a 3 Tesla Philips Achieva MRI (R3, v3.2.1.0) (Philips Healthcare, Cleveland, OH, USA) with an 8-channel pediatric SENSE head-spine coil. Prior to the scan, subjects were anesthetized with ketamine (15 mg/kg, IM), then intubated, immobilized using isoflurane (2‒2.5%) inhalation, positioned supine on the scanner bed and monitored during anesthesia. For consistency of positioning, and to prevent motion, the animals’ heads were immobilized inside the pediatric SENSE head-spine coil using soft pads. Special care was taken to make sure head positioning was consistent between animals, as well as between the different scanning sessions of the same animals. This stability was confirmed using the scout views prior to initiation of the scanning.

For the animals in Group A, T1 maps were generated using a dual-flip-angle method^28^. For this, two FFE images (60 slices, field of view (FOV): 110 ×110 x 60 mm, slice thickness: 1.0 mm, matrix: 220 × 220) were acquired in an axial orientation using a dual-flip angle SPGR protocol (TR/TE/FA1/FA2 = 34 ms/3.4 ms/5^o^/32^o^), both pre- and post-contrast. A phantom was placed in the FOV to serve as a normalization factor in T1 estimation of post-contrast scans. For this part of the study, Gadovist® (Gadobuterol, Bayer AG, Germany) was administered intravenously at 0.1 ml/kg. The timing of contrast administration and initiation of imaging was kept strictly constant across all animals and all scans of the same animals.

Accuracy and precision of T1 relaxation times’ measures from the dual-flip-angle method were assessed using gel phantoms (Leeds Test Object, North Yorkshire, UK) and a healthy NHP. First, an inversion recovery technique used to scan the phantoms provided measures within the estimated accuracy reported by the manufacturer for each phantom and was used as a ground truth. The phantoms were scanned at different times, days, and orientations and the coefficient of variance with respect to the range typical for white and gray matter was measured. Finally, uninfected NHPs were scanned multiple times within 1 day and the coefficient of variation of T1 values within ten selected regions of interest in the brain was measured.

For the animals in group B, FDG PET/CT scans were performed with a Philips Gemini PET/CT scanner (Philips Healthcare, Cleveland, OH, USA). Prior to the scan, subjects were anesthetized with ketamine (15 mg/kg, IM), then intubated, immobilized using isoflurane (2‒2.5%) inhalation, positioned supine on the scanner bed and monitored during anesthesia. CT images were acquired in helical scan mode with the following parameter settings: 140 kVp, 250 mAs/slice, 3 mm thickness, 1.5 mm increment, 0.688 mm pitch, collimation 16×0.75 and 0.5 s rotation. Two CT images were reconstructed from the raw data. An initial CT image was reconstructed into a 600-mm diameter field-of-view (FOV), resulting in a pixel size of 1.17 mm, and slice spacing of 3 mm. This CT image was used to correct the PET images for photon attenuation. The raw CT data were reconstructed a second time into a diagnostic quality CT image by reducing the FOV size to 250 mm, which resulted in a pixel size of 0.352 mm, and a slice spacing of 1.0 mm. All CT images were reconstructed into a 512 × 512 matrix using the standard “B” filter. No contrast was given, and the subjects were freely breathing during the scan. The diagnostic quality scans were reviewed by an experienced neuroradiologist (DAH) to rule out any structural brain abnormalities. Following the CT scan, a static PET emission scan covering the head was performed (FDG dose = 2.16 ± 0.15 mCi). The uptake period was 50 min. The scanner provides an axial FOV length of 180 mm for a single bed position. The scanning time was 5 min for one field of view. PET data were acquired in list mode and reconstructed using Philips’ iterative, maximum-likelihood reconstruction algorithm (3D-RAMLA) (Extended Brilliance Workspace (EBW) PET/CT application suite v1.30 29-Nov-2006) into images with a 128 × 128 matrix size and 2 mm wide cubic voxels. To ensure quantitative accuracy, all reconstructed PET images were corrected for radioactive decay during the scan, uniformity, random coincidences, attenuation and scattering. Both PET and CT images were then sent for radiologic interpretation and analysis. For 9 out of 15 animals in group B, structural brain MR imaging was performed using 3D MPRAGE (TE: 4.715 ms, TR: 9.75 ms, matrix: 96 × 96 x 68 mm, slice thickness: 0.5 mm).

For the animals in group C, structural imaging was performed including the following sequences: Proton density/T2 weighted imaging using a multi-echo sequence (TE = 20, 40, 60, and 80 msec; in-plane resolution = 0.43 × 0.43 mm; slice thickness = 2 mm, TR = 3300 ms, flip angle = 90°, fat suppression using SPIR, NSA = 1, and FOV = 96 × 96 x 60 mm); Susceptibility-weighted imaging using a principle of echo shifting/R2*(PRES R2*; 3D, FFE, in-plane resolution = 0.5 × 0.5 mm, slice thickness = 0.5 mm using over-contiguous slices; TR/TE = 34/42 ms, NSA = 1, flip angle = 5^o^, and FOV = 96 × 96 x 60 mm); Pre and post contrast T2 FLAIR imaging (turbo spin echo (TSE) sequence, in-plane resolution = 0.5 × 0.5 mm, slice thickness = 1 mm, TR = 10,000 ms, TE = 100 ms, NSA = 1, fat suppression using SPIR, TSE factor = 9, and FOV = 125 ×125 mm). In those animals, Magnevist® (Gadopentetate dimeglumine, Schering, Berlin, Germany) was administered intravenously at 0.2 ml/kg.

### Image analysis

Analysis of imaging data was performed using MIM software, version 7.0.4 (Cleveland, Ohio). For Group A, the D99 brain atlas was warped to fit a T1-weighted scan that was co-registered to the T1 maps^69^. The accuracy of registration of the D99 atlas was confirmed for all animals prior to performing any additional analysis. Volumes of interest (VOIs) including the whole brain as well as the frontal cortex, centrum semiovale, caudate, putamen, thalamus, hippocampus, midbrain, pons, medulla oblongata, and cerebellar white/gray matter were delineated on the D99 atlas. The VOIs were subsequently contracted circumferentially (range of 0.4–0.6 mm in all directions) to avoid partial averaging with CSF containing structures. The VOIs were then overlaid on co-registered pre- and post-contrast T1 maps. The phantom T1 values were used to normalize the post-contrast T1 values before calculating the percent change in T1 after contrast administration. Using these data, we obtained T1 pre-contrast values for each subregion demarcated by the D99 atlas. We also calculated percentage post-contrast T1-shortening (%T1-shortening) for each region by subtracting the post-T1 value from the pre-T1 value and dividing the product by the pre-T1 value ((Pre-Post)/Pre)*100%).

For Group B, the CT, MRI, and PET scans for a given scanning session were co-registered to one another. The same VOIs as above were automatically placed on the MRI brain by using the D99 template/atlas and then applied to the co-registered PET scan^69^. Contracting the VOIs circumferentially, as described above, helped decrease the potential for spill in/spill out (partial volume effect). The appropriate location of the VOIs following co-registration was checked prior to performing the analysis. VOIs included the whole brain as well as the regional VOIs (frontal cortex, centrum semiovale, caudate, putamen, thalamus, hippocampus, medulla, midbrain, pons and cerebellar white/gray matter). For subjects without MRI scans, an age- and weight-matched animal’s brain MRI was used to more accurately place the VOIs onto the PET scan.

Once the VOIs were placed, the corresponding average Standardized Uptake Values (SUVmean) were calculated as follows (1):

1 $${\rm{SUVmean}}=\frac{{\rm{Regional}}\; {\rm{radioactivity}}\; {\rm{concentration}}}{{\rm{Administered}}\; {\rm{radiotracer}}\; {\rm{dose}}/{\rm{Body}}\; {\rm{weight}}}$$

We then calculated relative SUV values by normalizing regional FDG uptake to whole brain FDG uptake, as follows (2):

2 $${\rm{RelativeSUV}}=\frac{{\rm{Regional}}\; {\rm{SUVmean}}}{{\rm{Whole}}\; {\rm{brain}}\; {\rm{SUVmean}}}$$

### Histology

In Group A, tissue samples from the cortex/striatum region, thalamic region and brainstem were obtained from nine animals euthanized at various time points post-infection. In addition, tissues from one uninfected animal were also obtained. Hematoxylin and eosin (H&E) stains were performed on the sections. Multiplex fluorescence immunohistochemistry (MF-IHC) staining was performed on brainstem and thalamic tissue sections for Iba1 (macrophages and microglia marker/ 1:200, Cedarlane labs #234006(SY)), CC3/PARP1 (cleaved caspase-3 and Poly [ADP-ribose] polymerase 1 markers as cell death markers/ 1:100, abcam #ab2302 and #ab32064), VP40 (a matrix protein found in EBOV/ 1:100, IBT bioservices #0201-17) and NeuN (neuronal marker/ 1:200, Millipore-Sigma #ABN90P). We also stained for albumin (to detect protein leakage into the brain in association with BBB disruption /1:100, Millipore-Sigma #A6684), CD31/CD144 (endothelial cell markers/ 1:100, abcam #ab9498 and #ab166715) and for glucose transporters, GLUT1 (1:100, abcam #ab40084) and GLUT3 (1:100, abcam #ab136180) to detect possible upregulation of glucose transporters in microglia and neurons. A more limited panel using NeuN (neuronal marker/ 1:200, Millipore-Sigma #ABN90P) and albumin (1:100, Millipore-Sigma #A6684) stains was used for the cortex/striatum slides. The cell nuclei were counterstained using 1 µg/ml DAPI to facilitate cell counting. All fluorescence signals were imaged using an Axio Imager.Z2 upright scanning wide-field fluorescence microscope (Zeiss) equipped with an Orca Flash 4.0 high-resolution sCMOS camera (Hamamatsu), 200 W X-cite 200DC broadband light source (Lumen Dynamics), and standard DAPI and Alexa Fluor filter sets (Semrock). After imaging, Zeiss Zen 3.0 software (Carl Zeiss Microscopy GmbH, Germany) was used for image acquisition, processing and stitching and Adobe Photoshop version 21.2.4 (Adobe, San Jose, California) was used for image visualization.

Quantification of the immunofluorescent staining was performed using FIJI image processing package, based on ImageJ, version 1.8.0 (NIH, Bethesda, MD). The RGB bitmap images were first converted to 8-bit grayscale and the threshold adjusted to include only cells of interest and eliminate the background. This was followed by calculating the fluorescence intensity within carefully selected regions of interest (ROIs). Since the stained regions were slightly heterogeneous due to slight variability in the orientation of the slides, about 70–100 small fields of view were carefully selected to provide consistency for quantifying staining across all animals. The data for all stains is expressed as percent fluorescence intensity relative to the area of the ROI. We also performed a visual evaluation of various neuronal cell bodies to assess for the percentage of VP40-positive cells compared to VP40-negative cells and to determine whether some neurons showed apoptotic markers without being VP40 positive. Besides measuring Iba1 fluorescent intensity, we looked for early subtle signs of activation such as increasing size of microglial cell body, and shortening/thickening of microglial processes. Finally, for each animal, we randomly selected six ROIs of the same size from different sections of the brainstem slides. We then manually delineated 12 cell bodies in each ROI, using Image J free hands selection tool, for a total of 72 cells per animal. The average size of the microglial cell bodies was then estimated and reported in area (pixels).

### Statistics

In order to decrease the effect of sepsis and multi-organ failure on our results, we eliminated imaging data obtained in the late stage of the disease (days 7 and 8) for all analyses. Statistical analysis was performed using S-plus software, version 8.2 (TIBCO software Inc, Palo Alto, California). The lme4 package^70^ (Code = lme), a built-in function in S-plus 8.2, was used.

For Group A, linear mixed models (LMM) were used to evaluate the effect of time post-infection (independent variable) on T1 pre-contrast values and %T1-shortening (dependent or outcome variables) in each VOI. Linear mixed-model analysis can determine a significant overall effect of time on T1 values and %T1-shortening and measure the magnitude and significance of the relationship between specific time points and changes in those values. Data from sequential days were grouped (Baseline, Day 1, Day 2/3, Day 4/5, Day 6) to increase the sample size of each time point. Random intercepts were used. Similar LMMs were used to evaluate the effect of time post infection (independent variable) on CSF cytokine levels (dependent variables). Significance was determined as *p*-value <0.01 to avoid type I errors. Significance trends were assigned for *p*-values between 0.01 and 0.05.

Additional LMMs were created to test for correlations between MRI-derived measures (T1 and %T1 shortening as dependent variables) and CSF cytokines, CSF viral load, and plasma viral load (independent variables). Biomarker values were transformed by taking the log10 values in cases where it produced a more normal distribution of data. Significance was determined as *p*-value < 0.01 to avoid type I errors.

For Group B, analysis was performed with both absolute and relative mean SUVs. Relative SUVs were calculated by normalizing the SUVs of regional VOIs to the SUV of the whole brain to detect regional differences in the setting of global hypo- or hypermetabolism. In order to test for changes in FDG uptake over the course of disease, LMMs were constructed with SUV or relative SUV as the dependent variable and time as the independent variable to determine whether there was a significant overall effect of time on SUV and to measure the magnitude and significance of the relationship between specific time points and changes in FDG uptake. For this analysis, data from sequential days were grouped (Baseline, Days 1/2, Days 4/5, Day 6) to increase the sample size of each time point. Similar LMMs were used to evaluate the effect of time post infection (independent variable) on plasma cytokine levels (dependent variables). Significance was determined as *p*-value < 0.01 to avoid type I errors. Significance trends were assigned for *p*-values between 0.01 and 0.05. Linear mixed models also assessed the relationships between FDG uptake (dependent variables) and other markers of disease including viral load, plasma cytokine levels, and measures from complete blood counts (independent variables). Biomarker values were transformed by taking the log10 values in cases where it produced a more normal distribution of data. Significance was determined as *p*-value < 0.01 to avoid type I errors.

### Reporting summary

Further information on research design is available in the Nature Research Reporting Summary linked to this article.

## Supplementary information

Supplementary Information

Reporting Summary

## Data Availability

Data supporting the findings of this work are available within the paper and its Supplementary Information files. A reporting summary for this Article is available as a Supplementary Information file. All additional raw data supporting the article or its Supplementary Information files are available from the corresponding author upon reasonable request (due to very large file sizes and animal welfare concerns) and will be provided through a data-sharing agreement directly with the user. Source data are provided with this paper.

## Source data

Source Data
