# Supplementary material for: Longitudinal in vivo imaging of acute neuropathology in a monkey model of Ebola virus infection
Source: Nat Commun. 2021 May 17;12:2855. doi: 10.1038/s41467-021-23088-x (PMC8129091; doi:10.1038/s41467-021-23088-x)
Supplement: Supplementary file 1 — Supplementary Information [file 41467_2021_23088_MOESM1_ESM.pdf]

Supplementary Information for

Longitudinal in vivo imaging of acute neuropathology in a monkey model of Ebola infection

Authors: William Schreiber-Stainthorp[1], Jeffrey Solomon[2], Ji Hyun Lee[3], Marcelo Castro[3], Swati Shah[1], Neysha Martinez-Orengo[1], Rebecca Reeder[3], Dragan Maric[4], Robin Gross[3], Jing Qin[5], Katie R. Hagen[3], Reed F. Johnson[6], Dima A. Hammoud[1*]

*Corresponding author. Email: hammoudd@cc.nih.gov

The PDF file includes:

Supplementary Figures

Supplementary Figure 1: Plasma cytokine levels in infected animals.

Supplementary Figure 2: CSF cytokine levels in infected animals.

Supplementary Figure 3. EBOV viral titers in various regions of the brain in group A animals.

Supplementary Figure 4: Appropriate co-registration of D99 atlas to brain MRI images

Supplementary Figure 5: Structural MR imaging at baseline and on day 6 post inoculation

Supplementary Figure 6: Multiplex fluorescence immunohistochemistry in the brainstem of control and infected animals

Supplementary Figure 7: Multiplex fluorescence immunohistochemistry staining for CC3/PARP1, NeuN, and GLUT3 in the brainstem of an infected animal.

Supplementary Figure 8: Multiplex fluorescence immunohistochemistry staining for Iba1 in the brainstem of control and infected animals.

Supplementary Tables

Supplementary Table 1: Changes in blood counts and viremia over time for groups A and B combined.

Supplementary Table 2: Changes in plasma cytokine levels (log10) over time for or groups A and B combined.

Supplementary Table 3: Changes in CSF cytokines over the course of disease for group A.

Supplementary Table 4: Correlations of imaging findings with disease biomarkers.

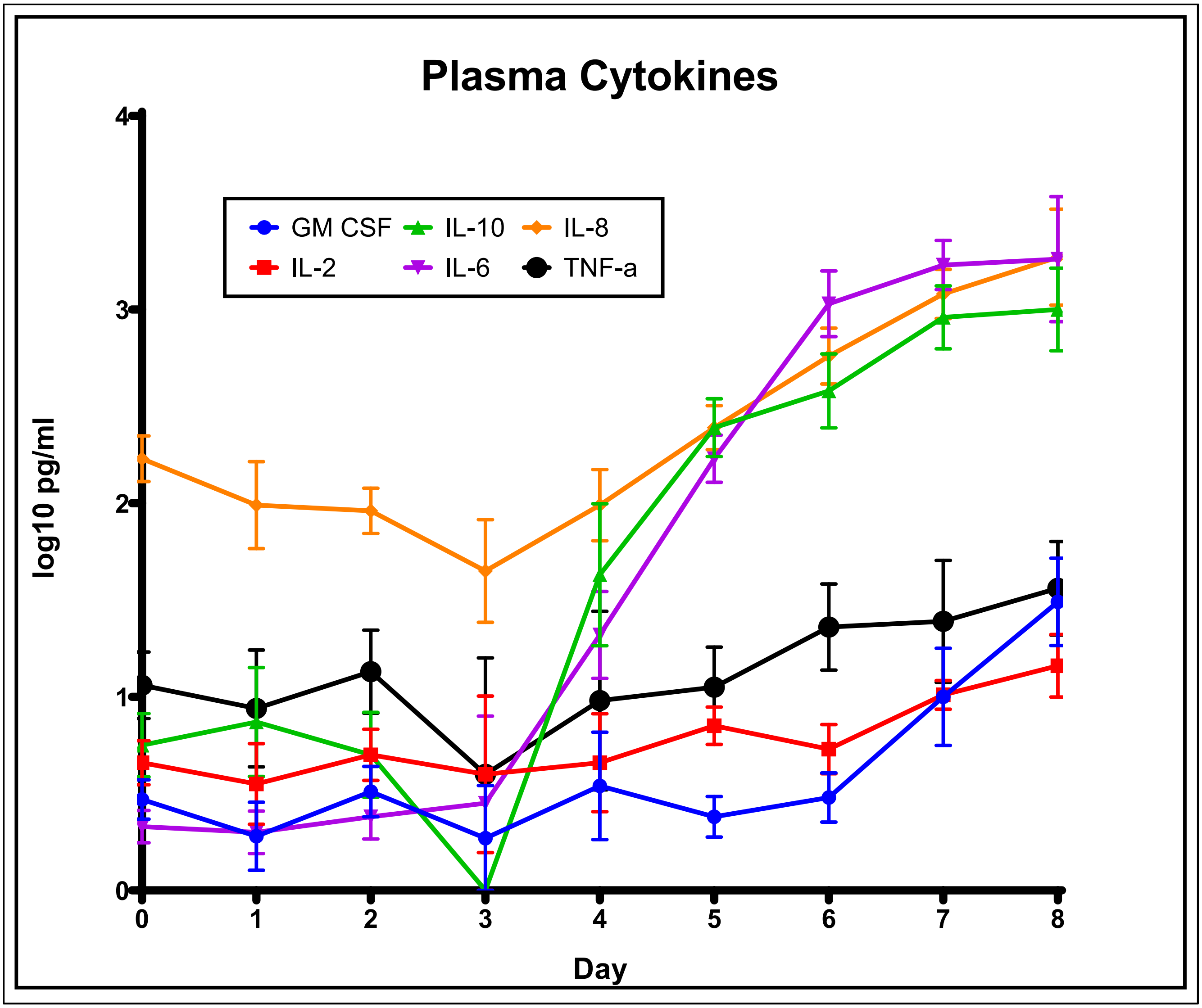

Supplementary Fig. 1. Select plasma cytokine levels of all infected animals (n=25). The increases over the course of the disease were statistically significant for IL-2, IL-6, IL-8 and IL-10 (Linear mixed effect model, S-plus 8.2, p= 0.0053, 8.47 e-12, 0.0019 and 7.98 e-8 respectively). Data are presented as mean values +/- SEM. Source data are provided as a Source Data file.

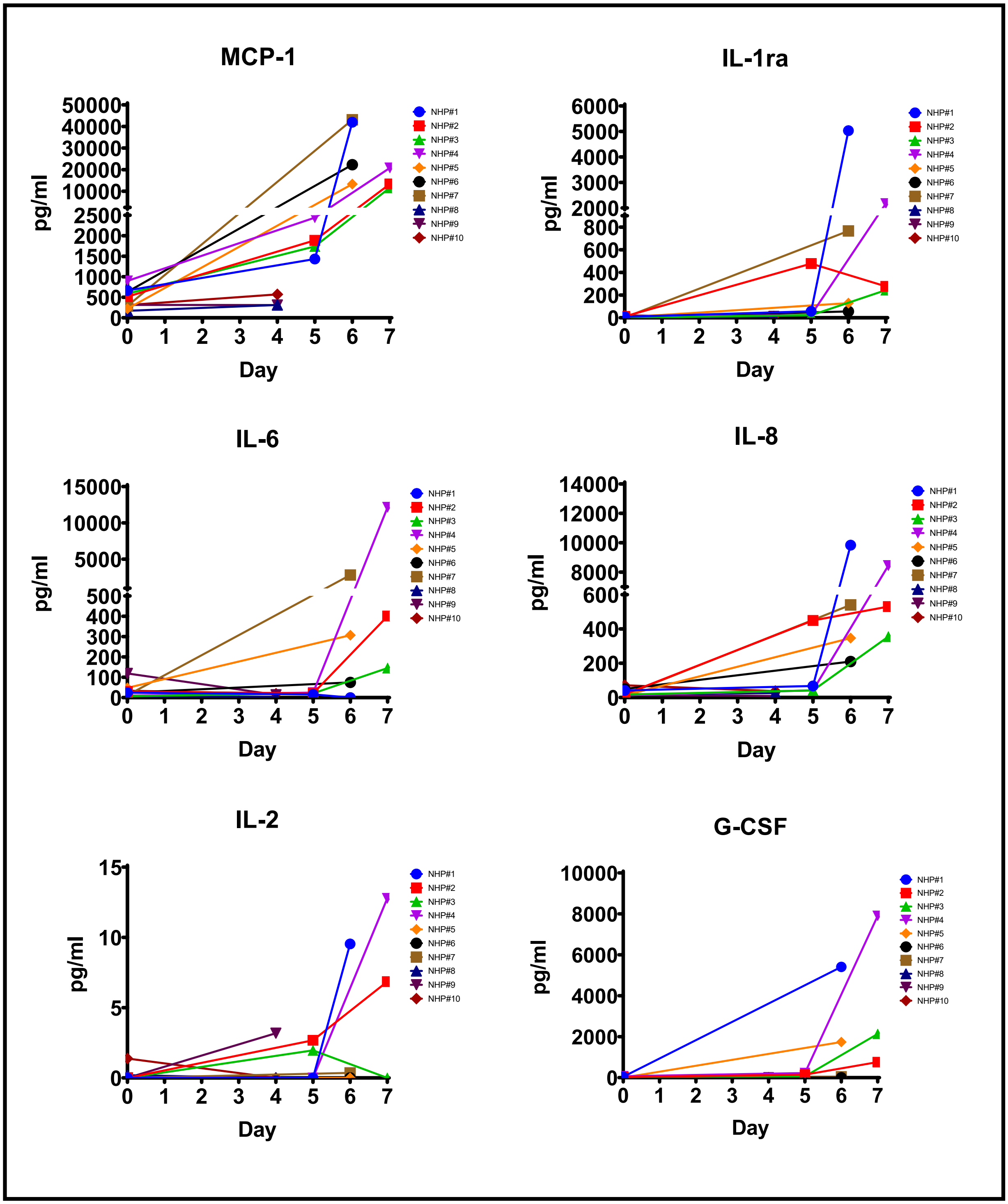

Supplementary Fig. 2. Select CSF cytokine levels of group A animals (n=10). The increases in CSF cytokine levels over time were statistically significant for MCP-1, IL-1Ra, IL-6 and IL-8 (Linear mixed effect model, S-plus 8.2, p=0.000013, 0.0016, 0.0041 and 0.0041 respectively). Source data are provided as a Source Data file.

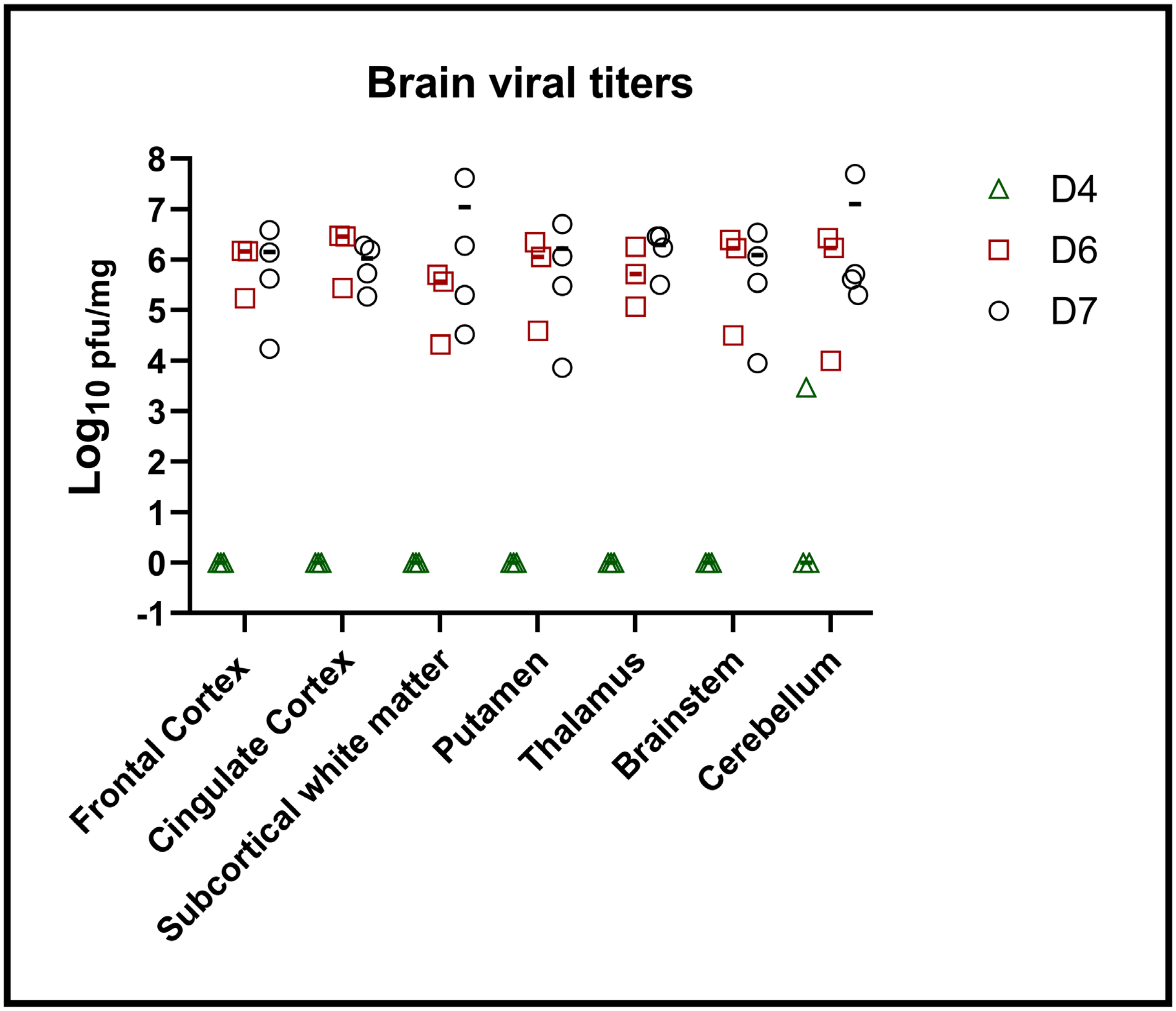

Supplementary Fig. 3. EBOV viral titers in various regions of the brain in group A animals (n=10 animals). Bars reflect mean values. Source data are provided as a Source Data file.

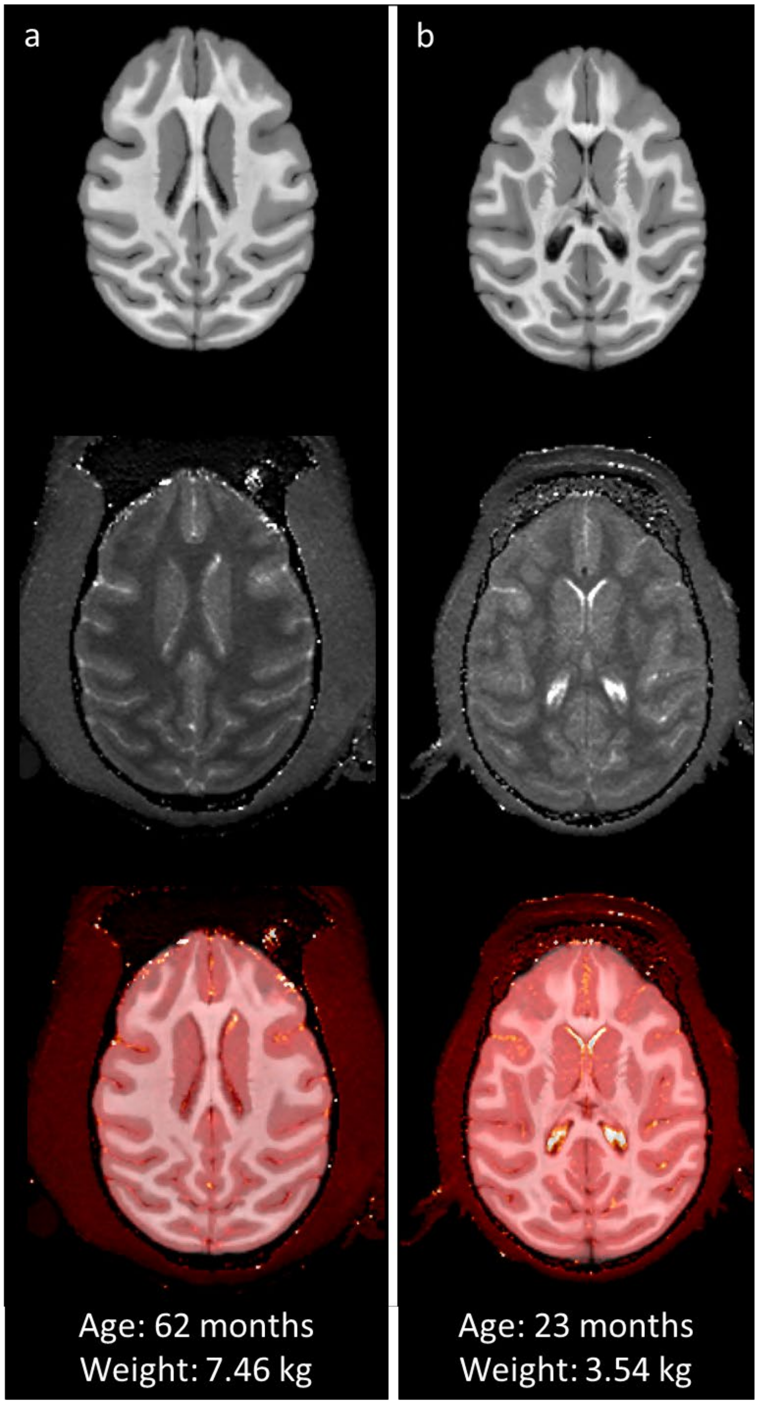

Supplementary Fig. 4. Appropriate co-registration of D99 atlas to brain MRI images shown for two animals aged 62 and 23 months, despite differences in age, weight and head size.

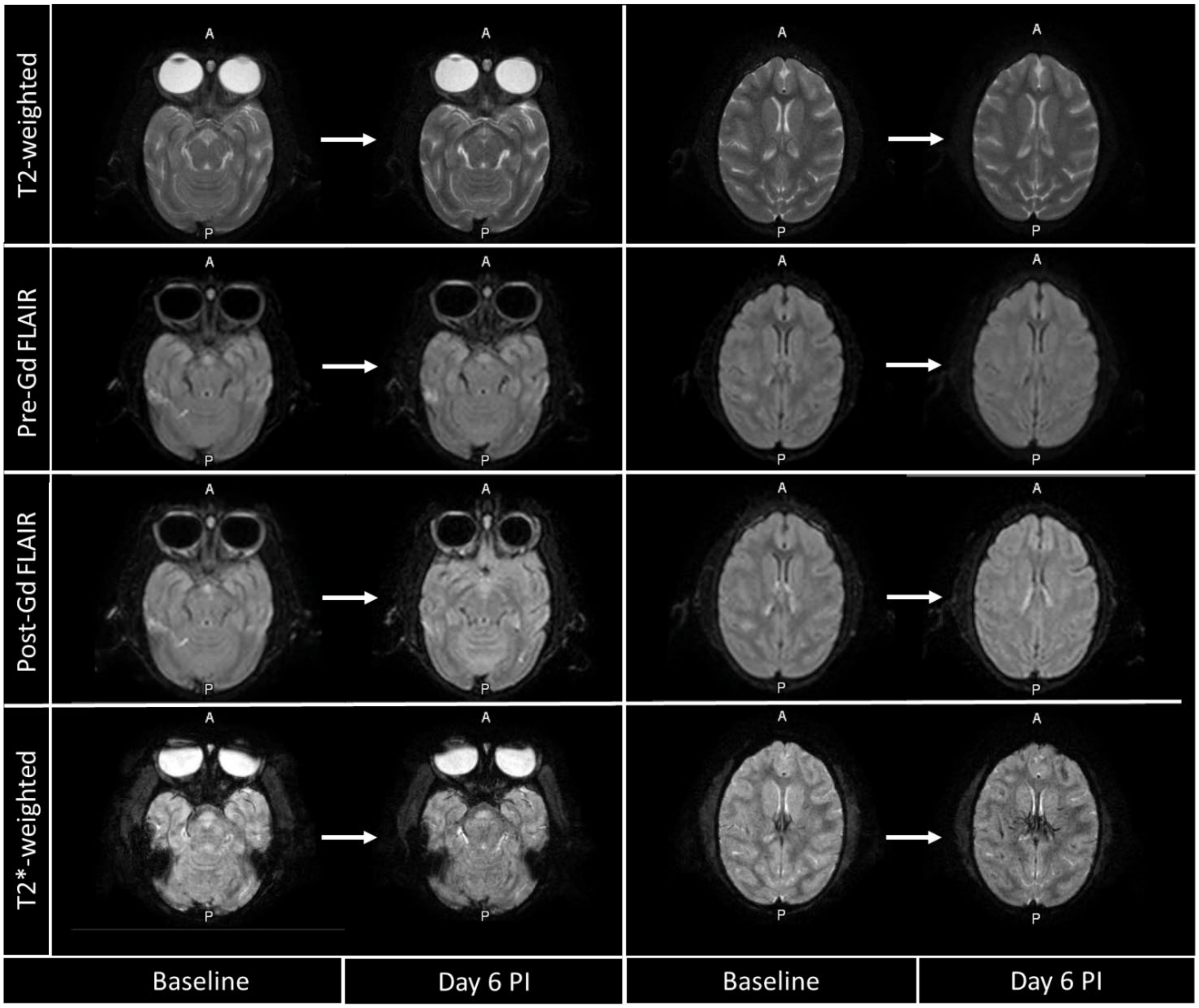

Supplementary Fig. 5: Structural MR imaging at baseline and on day 6 post inoculation showing no significant structural differences on T2-weighted, pre-contrast FLAIR or post contrast FLAIR images. Slightly increased prominence of central and cortical venous structures is seen on day 6 compared to baseline scans. Imaging was performed for two infected at baseline and day 6 post inoculation.

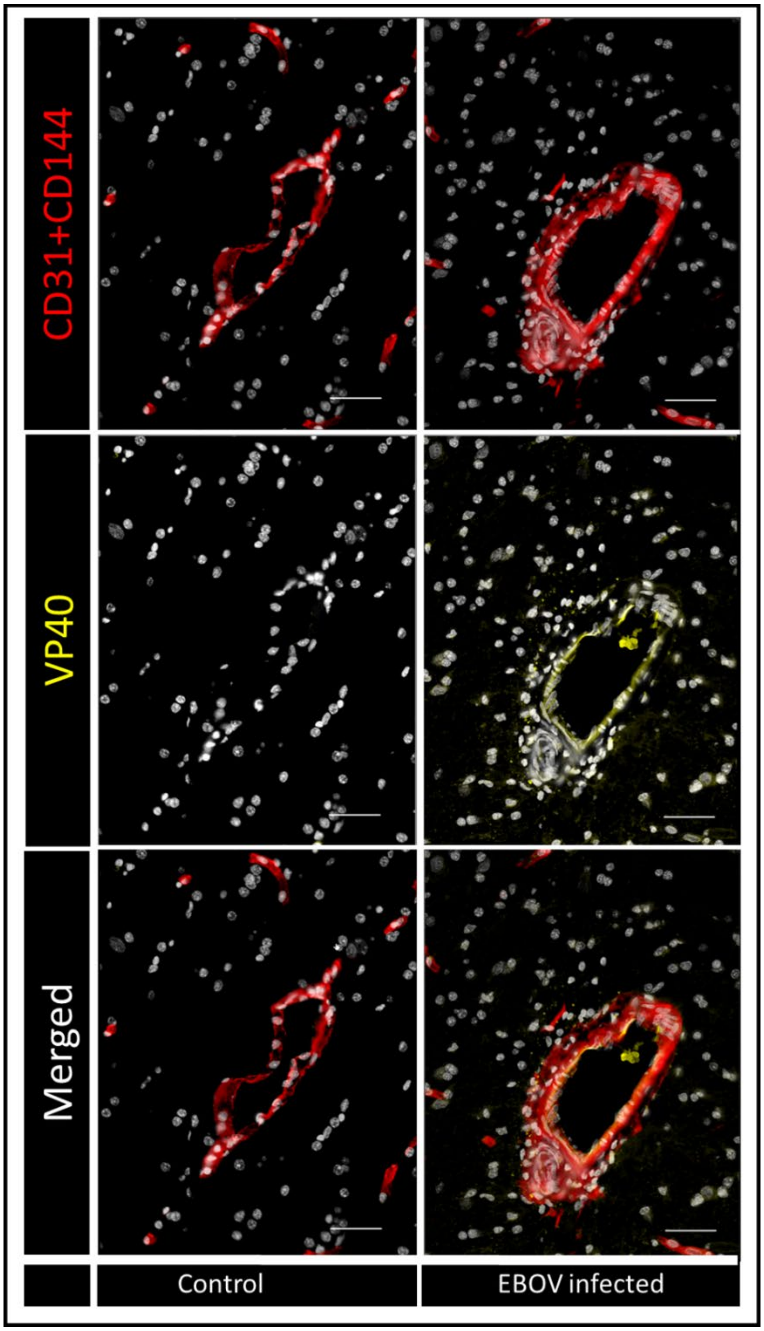

Supplementary Fig. 6: Multiplex fluorescence immunohistochemistry staining for VP40 and CD31+CD144 in the brainstem of control and infected animals. Multiplex fluorescence immunohistochemistry staining for VP40 (EBOV viral antigen, yellow) and CD31+CD144 (endothelial markers, red), in the brainstem of control (left) and infected (right) animals. VP40 staining is seen overlapping with endothelial markers in the infected animal. Staining was performed for nine infected and one control animal. Scale bar = 50 μm, magnification 150%

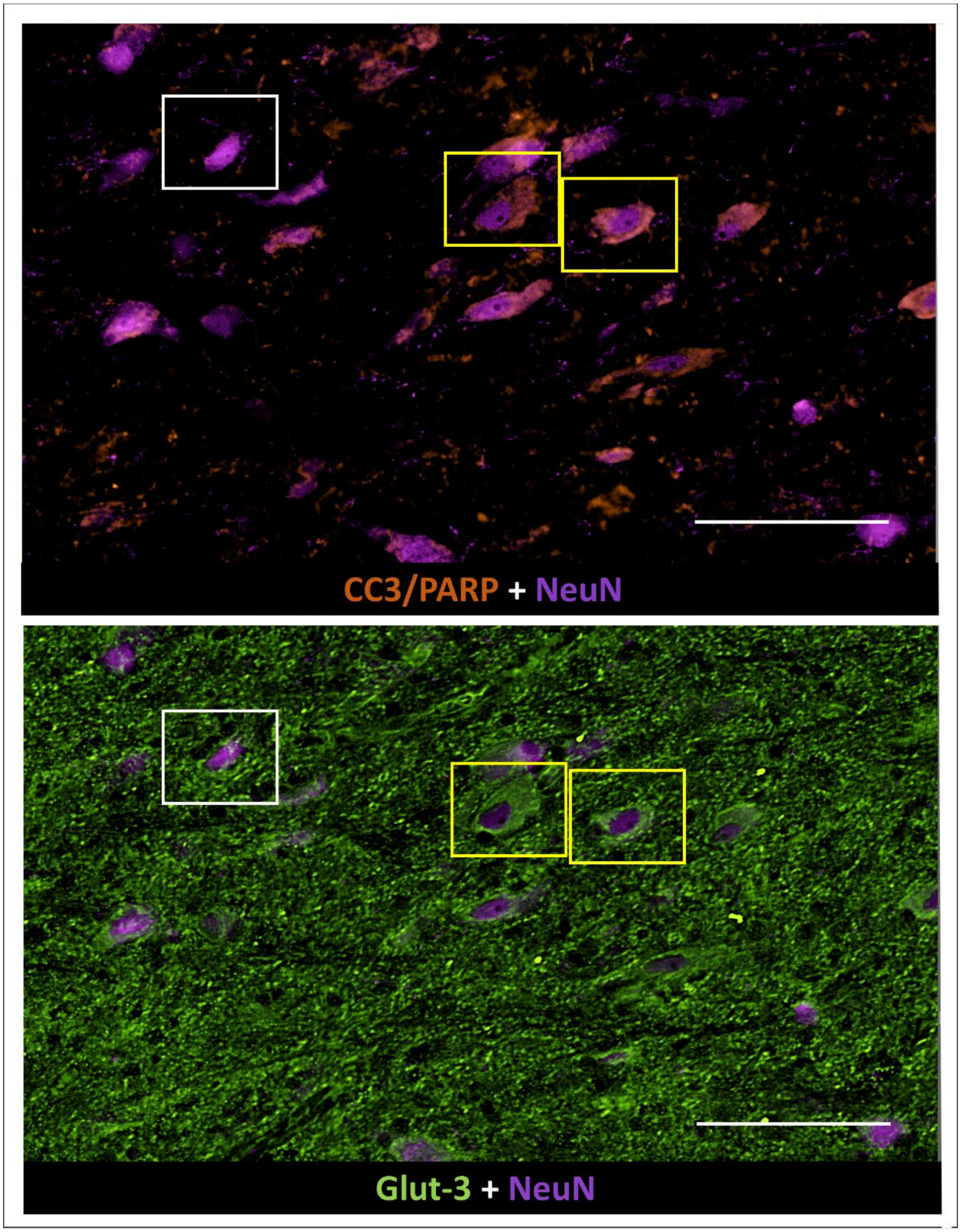

Supplementary Fig. 7: Multiplex fluorescence immunohistochemistry staining for CC3/PARP1 (apoptosis markers, orange), NeuN (neuronal marker, purple), and GLUT3 (glucose transporter, mainly expressed in neurons in the brain, green) in an infected animal. Unlike non-apoptotic neurons (white squares), apoptotic neurons (yellow squares) show increased GLUT3 staining. Staining was performed for nine infected and one control animal. Scale bar = 100 µm, magnification 100%

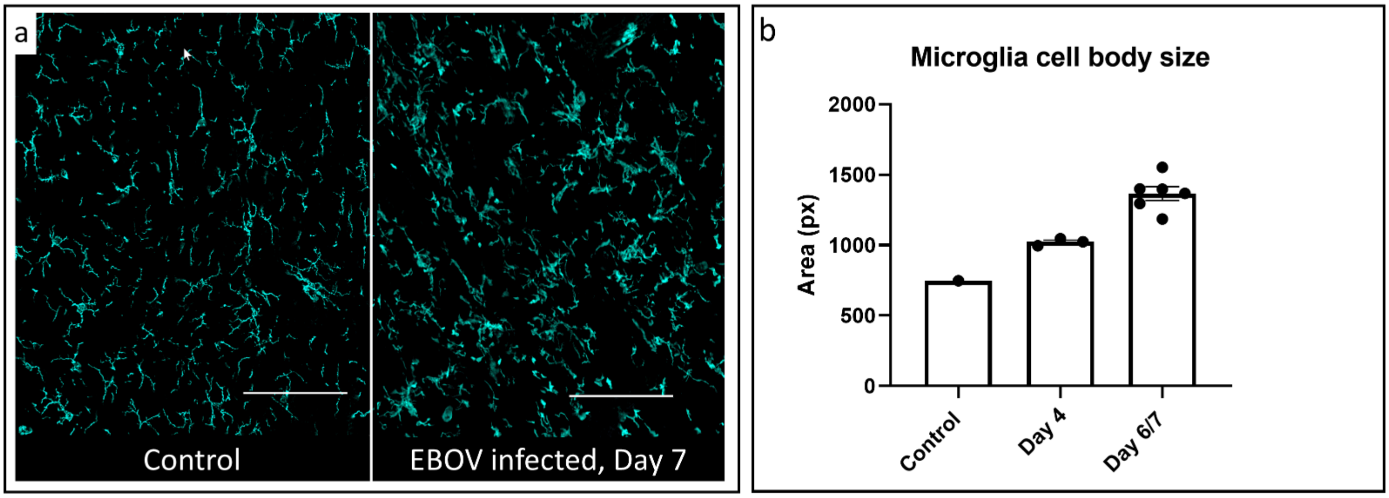

Supplementary Fig. 8: Multiplex fluorescence immunohistochemistry staining for Iba1 in the brainstem of control and infected animals. a. Signs of microglial activation are seen including slightly increased size of the microglial cell bodies with shortened and thickened processes. Scale bar = 100 µm, magnification 100%. Staining was performed for nine infected and one control animal. b. Microglial cell body size estimation in control (n=1) and infected animals (n=9). Data are presented as mean values +/- SEM. Source data are provided as a Source Data file.

Supplementary Table 1. Changes in blood counts and viremia over time for groups A and B combined.

Biomarker	Days Post-Infection									
	**Baseline**	**0**	**1**	**2**	**3**	**4**	**5**	**6**	**7**	**8**
Lymphocytes	3.8 (1.1)	3.1 (0.9)	2.7 (1.0)	3.3 (0.9)	2.5 (0.5)	1.7 (0.8)	1.1 (0.3)	1.5 (0.9)	1.6 (0.9)	0.1 (0.2)
Lymphocyte %	42.0 (10.9)	41.5 (12.2)	34.6 (8.7)	37.2 (13.2)	32.6 (7.4)	17.4 (11.6)	7.2 (2.7)	12.2 (7.2)	10.2 (5.0)	12.7 (5.7)
Platelets	385 (74)	394 (87)	370 (74)	392 (84)	467 (200)	329 (42)	256 (57)	173 (70)	114 (44)	91 (25)
Neutrophils	4.8 (1.9)	4.1 (2.2)	4.5 (1.3)	5.2 (2.0)	4.6 (0.9)	8.3 (3.7)	14.7 (5.6)	11.1 (4.6)	14.5 (5.0)	9.9 (7.0)
Neutrophil %	50.7 (11.6)	50.0 (13.7)	57.4 (9.2)	55.2 (15.0)	59.9 (7.0)	73.3 (12.6)	89.4 (3.9)	85.9 (7.6)	88.7 (5.2)	85.4 (6.5)
Reticulocytes	0.045 (0.016)	0.052 (0.031)	0.052 (0.026)	0.051 (0.014)	0.065 (0.016)	0.038 (0.015)	0.037 (0.015)	0.024 (0.008)	0.013 (0.006)	0.015 (0.006)
Reticulocyte %	0.79 (0.35)	0.97 (0.68)	1.01 (0.53)	0.92 (0.26)	1.28 (0.47)	0.76 (0.32)	0.68 (0.26)	0.53 (0.19)	0.25 (0.12)	0.30 (0.11)
Monocytes	0.59 (0.20)	0.57 (0.23)	0.59 (0.24)	0.57 (0.15)	0.54 (0.23)	0.93 (0.22)	0.50 (0.26)	0.22 (0.29)	0.16 (0.07)	0.22 (0.24)
Monocyte %	6.4 (1.9)	7.2 (1.8)	7.3 (2.1)	6.3 (1.6)	7.2 (3.2)	8.6 (1.4)	3.3 (1.7)	1.7 (1.9)	1.0 (0.3)	1.5 (0.8)
WBC	9.3 (2.0)	7.9 (2.3)	7.8 (1.8)	9.1 (1.6)	7.7 (0.7)	11.0 (3.3)	16.3 (5.7)	12.9 (4.8)	16.4 (5.3)	11.7 (8.5)
RBC	5.8 (0.4)	5.5 (0.4)	5.3 (0.4)	5.5 (0.2)	5.2 (0.5)	5.1 (0.3)	5.4 (0.3)	4.6 (0.4)	4.9 (0.5)	4.8 (0.7)
Hemoglobin	13.3 (0.7)	12.7 (0.6)	12.5 (0.6)	12.5 (0.3)	11.8 (0.3)	12.0 (0.3)	12.6 (0.6)	10.9 (0.5)	11.2 (1.2)	11.1 (0.9)
Hematocrit	41.1 (2.3)	39.5 (2.0)	38.3 (1.8)	39.1 (1.0)	36.0 (1.8)	37.1 (1.0)	38.8 (2.4)	32.9 (1.7)	34.1 (3.7)	33.7 (2.0)
log10 Plasma Viremia	0 (0)	0 (0)	0 (0)	0 (0)	3.1 (2.7)	6.5 (0.4)	8.4 (0.9)	9.4 (0.8)	9.9 (0.4)	9.6 (0.7)
Log10 CSF Viremia (group A)	0 (0)	ND	ND	ND	ND	1.5 (2.7)	3.4 (3.9)	4.7 (3.3)	7.8 (1.5)	ND

Supplementary Table 2. Changes in plasma cytokine levels (log10) over time for or groups A and B combined.

Biomarker (log10; pg/ml)	Days Post-Infection									
	Baseline	0	1	2	3	4	5	6	7	8
GM-CSF	0.56 (0.49)	0.47 (0.51)	0.28 (0.43)	0.51 (0.52)	0.27 (0.47)	0.54 (0.68)	0.38 (0.38)	0.48 (0.36)	1.00 (0.71)	1.49 (0.39)
IL-2	0.81 (0.47)	0.66 (0.57)	0.55 (0.51)	0.70 (0.53)	0.60 (0.70)	0.66 (0.62)	0.85 (0.35)	0.73 (0.36)	1.01 (0.21)	1.16 (0.28)
IL-10	0.84 (0.86)	0.75 (0.82)	0.87 (0.69)	0.70 (0.88)	0 (0)	1.63 (0.90)	2.39 (0.54)	2.58 (0.54)	2.96 (0.46)	3.00 (0.37)
IL-6	0.38 (0.44)	0.33 (0.42)	0.30 (0.27)	0.38 (0.46)	0.45 (0.78)	1.32 (0.55)	2.23 (0.44)	3.03 (0.48)	3.23 (0.36)	3.26 (0.56)
IL-8	2.25 (0.52)	2.23 (0.59)	1.99 (0.55)	1.96 (0.47)	1.65 (0.46)	1.99 (0.45)	2.39 (0.41)	2.76 (0.42)	3.08 (0.36)	3.27 (0.43)
TNF-α	1.25 (0.82)	1.06 (0.86)	0.94 (0.74)	1.13 (0.86)	0.60 (1.04)	0.98 (1.13)	1.05 (0.75)	1.36 (0.63)	1.39 (0.89)	1.56 (0.42)

Supplementary Table 3. Changes in CSF cytokines over the course of disease for group A.

**Biomarker (pg/ml)**	**Days Post-Infection**				
	**Baseline**	**4**	**5**	**6**	**7**
GM-CSF	0.44 (0.39)	0.86 (0.09)	0.58 (1.15)	0.49 (0.34)	0 (0)
TGF-alpha	4.46 (2.21)	2.78 (0.29)	6.16 (1.04)	6.16 (3.80)	19.94 (22.74)
G-CSF	18.85 (24.25)	1.14 (1.97)	146.16 (81.33)	1801.84 (2536.61)	3600.30 (3790.81)
IFN-γ	6.25 (19.33)	0.09 (0.11)	0.77 (1.33)	0.69 (1.23)	2.00 (2.73)
IL-2	0.16 (0.43)	1.06 (1.84)	1.16 (1.37)	2.47 (4.71)	6.54 (6.40)
IL-10	1.59 (3.88)	0 (0)	15.47 (22.13)	4.76 (9.51)	9.64 (6.97)
IL-15	13.80 (4.07)	14.19 (2.93)	14.10 (0.85)	29.00 (12.60)	61.48 (37.72)
IL-1Ra	6.34 (5.76)	10.76 (0.39)	145.51 (221.94)	1495.85 (2378.26)	897.76 (1104.33)
IL-13	0.40 (1.27)	3.46 (5.99)	1.15 (1.33)	0 (0)	0 (0)
IL-1b	0.01 (0.02)	0.05 (0.08)	0.53 (0.64)	25.55 (51.11)	11.47 (19.50)
IL-4	0.48 (1.52)	0 (0)	1.20 (2.40)	0 (0)	0 (0)
IL-6	31.17 (33.78)	12.15 (7.73)	20.66 (3.68)	800.53 (1352.86)	4236.01 (6864.59)
IL-8	27.47 (20.01)	24.07 (14.14)	150.13 (200.16)	2733.70 (4737.92)	3110.02 (4620.13)
MIP-1α	2.71 (2.35	4.74 (0.69)	1.81 (2.35)	9.20 (12.40)	0 (0)
MCP-1	460.78 (235.46)	397.78 (150.41)	1876.13 (421.77)	30169.53 (14757.74)	15252.07 (4888.86)
TNF-α	2.15 (6.81)	0 (0)	3.41 (6.83)	50.06 (100.13)	20.94 (36.27)
MIP-1β	0 (0)	0 (0)	0.58 (1.15)	1.52 (3.03)	0 (0)
IL-12/23 (p40)	0.21 (0.45)	0 (0)	2.36 (4.72)	0.88 (1.77)	6.29 (5.46)
IL-18	0 (0)	0 (0)	6.05 (6.99)	81.54 (127.17)	74.51 (127.76)

Supplementary Table 4: Correlations of imaging findings with disease biomarkers. **a.** Significant correlations of T1 values with disease biomarkers (n=10 animals, Linear mixed effect model, S-plus 8.2). **b.** Significant correlations of post contrast %T1 shortening with disease biomarkers (n=10 animals, Linear mixed effect model, S-plus 8.2). **c.** Significant correlation of FDG relative SUV values with disease biomarkers (n=15 animals, Linear mixed effect model, S-plus 8.2). Estimated coefficients (EC) are included.

a

T1 values	Centrum semi ovale	Frontal cortex	Caudate	Putamen	Thalamus	Cerebellum GM
Plasma viral load	--	--	P= 0.005 (EC=0.002)	P= 0.001 (EC=0.002)	--	--
CSF Viral load	--	--	--	--	--	--
GM CSF	--	P= 0.008 (EC=0.032)	--	--	--	--
MIP-1α	--	P= 0.0009 (EC=0.026)	--	--	--	--

b

% T1 shortening	Centrum semi ovale	Frontal Cortex	Caudate	Putamen	Thalamus	Cerebellar GM
Plasma viral load	--	--	--	P= 0.006 (EC=0.294)	P= 0.002 (EC=0.403)	P= 0.004 (EC=0.331)
CSF Viral load	--	P= 0.008 (EC=0.646)	--	--	--	P= 0.009 (EC=0.611)
IL-1ra	--		--	--	--	P=0.006 (EC=2.518)
IL-1β	--	P=0.004 (EC=5.254)	--	--	--	
IL-8	--	--	--	--	--	P=0.006 (EC=3.816)
IL-18	--	--	--	--	--	P= 0.003 (EC=2.315)
GM-CSF	--	P= 0.009 (EC=3.674)	--	--	--	--
MIP-1α	--	P= 0.0004 (EC=3.974)	P= 0.003 (EC=2.581)	P= 0.008 (EC=2.352)	--	--
MIP-1β	P= 0.0002 (EC=4.94)	P= 0.009 (EC=4.581)	--	--	--	--

c

FDG uptake	Relative SUV Thalamus	Relative SUV Midbrain	Relative SUV Pons	Relative SUV Medulla
Plasma Viral load	--	--	P=0.0002 (EC=0.018)	--
IL-8	P=0.0005 (EC=0.149)	P= 0.0004 (EC=0.192)	P= 0.00008 (EC=0.228)	P=0.001 (EC=0.239)
IL-6	P=0.003 (EC=0.030)	P=0.006 (EC=0.039)	P=0.00007 (EC=0.057)	P=0.003 (EC=0.059)
IL-10	--	--	P= 0.006 (EC=0.049)	--
